# Transcriptome Based Profiling of the Immune Cell Gene Signature in Rat Experimental Colitis and Human IBD Tissue Samples

**DOI:** 10.3390/biom10070974

**Published:** 2020-06-29

**Authors:** Éva Boros, Bence Prontvai, Zoltán Kellermayer, Péter Balogh, Patrícia Sarlós, Áron Vincze, Csaba Varga, Zoltán Maróti, Balázs Bálint, István Nagy

**Affiliations:** 1 Institute of Biochemistry, Biological Research Centre, 6726 Szeged, Hungary; boros.eva88@gmail.com (É.B.); prontvaibence@gmail.com (B.P.); 2 Department of Immunology and Biotechnology, University of Pécs, 7624 Pécs, Hungary; kellermayer.zoltan@pte.hu (Z.K.); balogh.peter@pte.hu (P.B.); 3 Lymphoid Organogenesis Research Group, Szentágothai János Research Center, University of Pécs, 7624 Pécs, Hungary; 4 1st Department of Internal Medicine, Medical School, University of Pécs, 7624 Pécs, Hungary; sarlos.patricia@pte.hu (P.S.); vincze.aron@pte.hu (Á.V.); 5 Department of Physiology, Anatomy and Neuroscience, University of Szeged, 6726 Szeged, Hungary; vacs@bio.u-szeged.hu; 6 Institute of Pediatrics and Pediatric Health Center, University of Szeged, 6720 Szeged, Hungary; zmaroti@gmail.com; 7 Seqomics Biotechnology Ltd., 6782 Mórahalom, Hungary; h836472@gmail.com

**Keywords:** inflammatory bowel disease (IBD), immune cell gene signature (ImSig), whole transcriptome analysis

## Abstract

Chronic intestinal inflammation is characteristic of Inflammatory Bowel Disease (IBD) that is associated with the exaggerated infiltration of immune cells. A complex interplay of inflammatory mediators and different cell types in the colon are responsible for the maintenance of tissue homeostasis and affect pathological conditions. Gene expression alteration of colon biopsies from IBD patients and an in vivo rat model of colitis were examined by RNA-Seq and QPCR, while we used in silico methods, such as Ingenuity Pathway Analysis (IPA) application and the Immune Gene Signature (ImSig) package of R, to interpret whole transcriptome data and estimate immune cell composition of colon tissues. Transcriptome profiling of in vivo colitis model revealed the most significant activation of signaling pathways responsible for leukocyte recruitment and diapedesis. We observed significant alteration of genes related to glycosylation or sensing of danger signals and pro- and anti-inflammatory cytokines and chemokines, as well as adhesion molecules. We observed the elevated expression of genes that implies the accumulation of monocytes, macrophages, neutrophils and B cells in the inflamed colon tissue. In contrast, the rate of T-cells slightly decreased in the inflamed regions. Interestingly, natural killer and plasma cells do not show enrichment upon colon inflammation. In general, whole transcriptome analysis of the in vivo experimental model of colitis with subsequent bioinformatics analysis provided a better understanding of the dynamic changes in the colon tissue of IBD patients.

## 1. Introduction

Inflammatory bowel disease (IBD) is an umbrella term of chronic inflammatory disorders of the gastrointestinal tract [1]. Its main types are Crohn’s disease (CD) and ulcerative colitis (UC), whose incidence is increasing worldwide but mostly concern westernized countries [2]. Symptoms of CD and UC influence the everyday life of patients, because disruption of intestinal homeostasis not only eventuate bloody diarrhea, bloating, abdominal pain and cramps, malabsorption and fatigue, but also narrow down social life and trigger depression [1,3]. As a remittent disorder, it is characterized by the fluctuation of nearly asymptomatic remission and symptomatic recurrence phases, when sporadically located inflamed lesions appear along the intestine [1].

The molecular background of IBD is extremely complex because of its multifactorial pathogenesis [4]. Previous studies described the effects of genetic alterations, environmental factors, life-style habits and the microbiome in the progression of IBD, but their independent examination may lead astray [4,5]. Whole genome association studies have established the importance of mutations affecting genes with diverse roles in the communication between host and microbiome, such as NOD2 or FUT2 [6,7,8]. Characterization of gut flora of healthy individuals and IBD patients is currently a popular field of research, but it has not been ascertained yet whether the alteration of microbiome is the cause or the consequence of the disease [9,10]. Heterogeneity in the manifestation of symptoms, genetic background or the microbiome between individual patients obstruct the efficient determination of a general master regulator in this multilevel system.

Accordingly, instead of focusing only on certain molecules or mutations, the comprehensive investigation of gene expression patterns is more informative in defining the consequences of the interactions between these various factors. Whole transcriptome analysis of an in vivo model of experimentally induced colitis represents a snapshot of the inflamed colon, and enables the simultaneous observation of thousands of mRNA molecules. In this work we examined different aspects and participants of inflammatory response by following an axis from the gut lumen through the network of tissue resident and infiltrating immune cells to the endothelial cells of the vascular wall, by the measurement of gene expression pattern of affected genes in rat and human colon tissue samples. Furthermore, we applied several bioinformatics tools in order to decipher more precisely the detected gene expression signatures. We determined the activated signaling pathways by applying Ingenuity Pathway Analysis (IPA); in addition, we used the ImSig package of R to define the immune cell composition of colon tissues based on gene expression signatures.

## 2. Materials and Methods

### 2.1. In Vivo Rat Model and Sample Collection

To investigate the molecular background of IBD we used the 2,4,6-trinitrobenzene sulfonic acid (TNBS) induced rat model of experimental colitis. Male Wistar rats (180–220 g) were randomly divided into two groups as detailed in [11]: the first group served as control, and the second group was induced by TNBS (colitis-induced) based on the method described by Morris et al. [12]. Seventy-two hours after the treatment, all animals were sacrificed and distal colons were removed. In the control group (*n* = 2), samples were taken from random colon sections, while samples from colitis-induced animals were taken from the inflamed colon region (*n* = 6) as well as from a non-adjacent uninflamed region (*n* = 6). All samples were kept in TRIzol reagent (Thermo Fisher Scientific, Waltham, United States) at −80 °C.

### 2.2. IBD Patients

Colonic biopsies were obtained from 21 consenting patients with IBD (Table 1, 13 females and eight males; median age 41 years, 25–74 years) undergoing colonoscopy for diagnostic purposes approved by Hungarian Medical Research Council (ETT, Budapest, Hungary) Medical Research Council’s Committee of Scientific and Research Ethics (TUKEB) as described previously [13]. Human colonic biopsies were obtained by experienced gastroenterologists at the 1st Department of Internal Medicine, University of Pécs in accordance with the guidelines set out by the Medical Research Council of Hungary. Sample collection and classification was performed according to disease status of patients, active/relapsing or inactive/remission phase. Furthermore, samples from relapsing patients were subdivided as uninflamed or inflamed according to the status of colon tissue. To classify uninflamed from inflamed samples, macroscopic diagnostic features were used in accordance with the recommendations of the European Crohn’s and Colitis Organisation [14]. Multiple samples were obtained both from visible lesions and from mucosa which is normal on gross inspection. Classically, the mucosa in UC has a friable granular appearance and shows superficial ulcers. The earliest visible mucosal lesions of CD are small aphthous ulcers, after which they coalesce to large deep serpiginous or linear ulcers.

### 2.3. Extraction of Total RNA

Samples from rat colons were homogenized in TRIzol reagent by ULTRA-TURRAX T-18 (IKA) instrument as described previously [15]. In total, 0.1 mL of chloroform (Sigma-Aldrich, Haverhill, United Kingdom) was added to 0.3 mL homogenized sample with vigorous vortexing. Samples were centrifuged at 13,000 rpm for 10 min. Total RNA was than extracted from the upper aqueous phase. RNeasy Plus Mini Kit (Qiagen, Hilden, Germany) was used to purify total RNA from rat colon samples according to the manufacturer’s protocol. Total RNA from human biopsies was isolated using NucleoSpin RNA Kit (Macherey-Nagel, Düren, Germany) according to the manufacturer’s protocol. The quality and the quantity of the extracted RNAs were determined by TapeStation (Agilent, Santa Clara, United States) and Qubit Fluorometer (Thermo Fisher Scientific).

### 2.4. Reverse Transcription and Quantitative Real-Time PCR (QPCR)

Reverse transcription was performed by SuperScript VILO Master Mix (Thermo Fisher Scientific) according to the manufacturer’s instructions. Gene expression was measured by quantitative real-time PCR using the StepOne PCR Systems (Thermo Fisher Scientific). SybrGreen technology based QPCR reactions were performed by SYBR Select Master Mix (Thermo Fisher Scientific) with specific exon spanning primer sets (Table 2 and Table 3), while TaqMan technology based reactions were performed by TaqMan Fast Advanced Master Mix and TaqMan Gene Expression Assays (Table 4) (Thermo Fisher Scientific). The ratio of each gene relative to the 18S rRNA was calculated using the 2^−ΔΔCT^ method.

### 2.5. Whole Transcriptome Analysis by RNA-Seq and Bioinformatics

**Figure 1:**
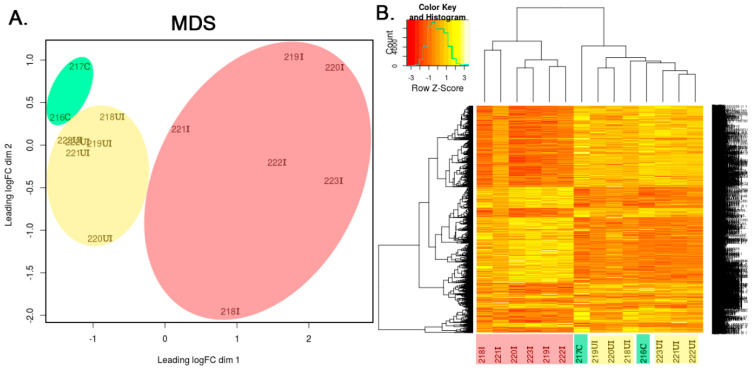
In silico performed multidimensional scaling (MDS) of individual transcriptomic data clustered samples into control, 2,4,6-trinitrobenzene sulfonic acid (TNBS)-treated uninflamed and TNBS-treated inflamed groups, while examination of significantly altered transcripts confirmed these observations. (**A**) MDS of individual rat colon samples and (**B**) Representative heatmap image of significantly altered transcripts in the control (green, *n* = 2), TNBS-treated uninflamed (yellow, *n* = 6), TNBS-treated inflamed (red, *n* = 6) tissues. The heatmap contains 3766 genes that displayed significant gene expression change in at least one of the three comparisons made. In the sample IDs, the abbreviations “C,” “UI” and “I” represent control, uninflamed and inflamed sample groups, respectively.

RNA-Seq was performed by SOLiD total RNA-Seq Kit (Thermo Fisher Scientific), according to the manufacturer’s instructions, and sequenced on SOLiD 5500xl instrument using the 50-base sequencing chemistry. Raw sequence data was size-selected discarding reads shorter than 50 bp. Rnor_5.0 (GCA_000001895.3) from ENSEMBLE release 77 was used as a reference to align RNA-Seq data. Legacy RNA-Seq engine of CLC Genomic Workbench tool (Qiagen, v7.5.1) was used to obtain gene expression estimates (mapped read counts) for each annotated gene in all samples with the following mapping parameters: maximum number of mismatches: 2, minimum alignment length fraction: 0.8, minimum similarity fraction: 0.8, unspecific match limit: 10. Reads mapping to rRNA genes were removed. Subsequently, “calcNormFactors” from package “edgeR” was used to perform data normalization based on the “trimmed mean of M-values” (TMM) method. Common and tagwise negative binomial dispersion values were estimated in edgeR using “estimateCommonDisp” and “estimateTagwiseDisp” functions of edgeR package, respectively. Lists of differentially expressed genes were obtained via the “exactTest” function of edgeR. After data normalization, genes showing at least two-fold gene expression change with a false discovery rate (FDR) value below 0.05 were considered significant. Multi-dimensional scaling was also applied to visually summarize gene expression profiles, revealing similarities between samples and unsupervised cluster analysis was carried out on the normalized data. Function plotMDS() from package Limma was used to generate Figure 1A. This tool performs multidimension scaling on the sample-sample distance data calculated from gene expression profiles. Distance between each pair of samples is determined as the root-mean-square deviation (Euclidean distance) for the top N genes (N = 500 in our analysis) that display the highest fold change between the given two samples. Such distance values can be interpreted as “leading log2-fold-change,” meaning the typical log2-fold-change of the top N genes that distinguish the compared two samples. Values in the x and y axis of the multidimensional scaling (MDS) plot also represent this distance calculation logic. The first two dimensions of the MDS scaling were calculated to account for 37.7% and 10.5% of the variance in data, respectively.

### 2.6. Pathway Analysis

For the interpretation of transcriptomic data and for the definition of functional relationship between significantly changed molecules, we used the Ingenuity Pathway Analysis (IPA) application (http://www.ingenuity.com). Database of IPA (Knowledge Base) is based on published experimental results. Significantly activated canonical pathways in the in vivo model of experimental colitis were determined by core analysis function of IPA by using log fold change and FDR values. Determination of significantly activated canonical pathways was based on two different methods. The most significantly altered canonical pathways between sample groups of RNA-Seq analysis were calculated using a Right-Tailed Fisher’s Exact Test; it reflects the likelihood that the association or overlap between a set of significant molecules from the experiment and given process/pathway transcription neighborhood is due to random chance. The smaller the *p*-value, the less likely that the association is random; for a clear visualization, values were transformed into negative log scale, where a higher “–log(*p*-value)” means it is less likely that the association is random. By using the downstream effect activation of the z-score, a statistical measure of the correlation between the relationship direction and the resulting gene expression were performed. Z-score > 2 or < −2 is considered significant, which can be applied in some analysis types, and provides predictions about upstream or downstream processes. It takes into account the directional effect of one molecule on another molecule or on a process, and the direction of change of molecules in the dataset.

### 2.7. Translation of Homologous Immune Signature Gene IDs

We used the Complete List of Vertebrate Homology Classes database of the Mouse Genome Database (MGD) [16] to identify the homologous *Rattus norvegicus* gene IDs of the *Homo sapiens* Immune Signature genes. Out of the 569 immune human signature genes, we found 476 IDs in the MGD database, and an additional 41 homolog/ortholog IDs were identified manually using the resources of NCBI and HGNC databases, while we did not find a rat counterpart of 52 human signature genes.

### 2.8. Immune Signature Analysis

According to the recommendations of the original manuscript [17], we used the prenormalized (calculated expression signal of a gene per sample) transcriptomic data from SOLiD 5500xl platform for this analysis. Normalization of gene expression data was performed by the TMM method of edgeR package (version 3.24.3) and a conservative correlation threshold (r = 0.7) was used for feature selection and immune signature analysis (ImSig R package, version 1.0.0).

### 2.9. Statistical Analysis, Data Representation and Data Availability

Statistical evaluations were performed using the IBM SPSS Statistics program for Windows. Graphs were plotted with GraphPad Prism 6 software or by ggplot package of R. Quantitative data are presented as the mean ± SEM and the significance of difference between sets of data was determined by one-way analysis of variance (ANOVA) following LSD post-hoc test; a *p* value of less than 0.05 was considered significant.

Gene Expression Omnibus (GEO) archive of the three sequenced libraries was deposited in NCBI’s GEO Archive at http://www.ncbi.nlm.nih.gov/geo under accession GSE149517.

## 3. Results and Discussion

### 3.1. Global Gene Expression Pattern of In Vivo Model of Colitis

In the widely used 2,4,6-trinitrobenzene sulfonic acid (TNBS) induced rat model of IBD the intracolonically-administered TNBS destroys the integrity of intestinal epithelium and generates sporadically located lesions [18]. Hence, inflamed, phenotypically normal and uninflamed tissue regions alternate along the colons, similar to that observed in the digestive tract of IBD patients [1]. To investigate the transcriptional changes after TNBS induction in different regions of the colon, we took samples from both the inflamed and the uninflamed tissue areas and compared them to untreated control samples using whole transcriptome analysis by RNA-Seq. After the standard processing of raw sequencing data by bioinformatics tools, as described in Section 2.5, multidimensional scaling (MDS) was performed to visually summarize gene expression profiles revealing similarities and differences between samples. In silico performed MDS of individual transcriptomic data demonstrated clustering of samples into control, TNBS treated uninflamed and TNBS treated inflamed groups (Figure 1A). In addition, heat map representation of significantly altered transcripts confirmed differences between inflamed and uninflamed samples of TNBS treated animals, whereas control and uninflamed samples proved to be very similar at global transcriptomic level (Figure 1B).

Based on two independent methods, TNBS treated uninflamed and inflamed regions of the colon significantly differ from each other at transcriptional level. To quantify the transcriptomic data, we compared gene expression values of sample groups to each other. The expression of ~3800 transcripts changed significantly in at least one of the three different ways of comparisons, namely TNBS treated –uninflamed vs. control (abbreviated as UI-C), TNBS-treated–inflamed vs. control (abbreviated as I-C) and TNBS treated-inflamed vs. TNBS treated–uninflamed (abbreviated as I-UI).

### 3.2. Correlation between Murine and Human Samples

To validate our transcriptome data, first we compared the RNA-Seq results to our previous data on gene expression changes related to epithelial-to-mesenchymal transition (EMT) [13,15,19]. Upon EMT, epithelial cells lose their epithelial characteristics, their cell-cell connections disintegrate and cell motility enhances. This process is associated with the formation of intestinal fibrosis and may play a role in the development of IBD-related colorectal cancer [20]. We have previously reported that the expression of EMT activating genes significantly increased both in the inflamed colon samples of TNBS treated rats as well as in IBD patients [13,15,19]. In addition, the expression of growth factors (EGR1, FGF2 and FGF7), signal transducer molecules (JAK2, NOTCH2 and HIF1α), EMT inducing transcriptional factors (ZEB2, SNAI1), extracellular matrix remodeler MMP9 and mesenchymal markers (LOX and VIM) are all upregulated in the inflamed colon tissues, while the expression of the epithelial marker CDH1 decreased [13,15,19]. Here, we report perfect overall correlation between the aforementioned QPCR measurements and our current RNA-Seq results for all 12 genes (Supplementary Figure S1), indicating that the RNA-Seq data are of high quality, thus allowing further analysis.

### 3.3. In Silico Analysis of Transcriptomic Data

In order to better understand results obtained by RNA-Seq and to identify possible relationship/s between molecules with altered expression, next, we performed in silico pathway analysis by using IPA. Functional analysis of our data sets revealed significant alteration of canonical pathways related to communication between immune cells, immune cell recruitment, signaling through pattern recognition receptors and regulation of inflammatory response at the site of inflammation; in line with this, we detected the activation of NF-κB signaling that interconnects several inflammation regulating pathways (Supplementary Tables S1 and S2). Based on the p-values, the most significantly activated pathways were the “Granulocyte adhesion and diapedesis,” “Agranulocyte adhesion and diapedesis” and “Leukocyte extravasation signaling” (Supplementary Table S1). By applying the z-score “Acute phase response signaling,” “IL6 signaling” and “Role of pattern recognition receptors in recognition of bacteria and viruses” pathways (Supplementary Table S2) were also highly enriched. The overview of these circuits represents the actual status of the rat colon in the TNBS-induced colitis; thus, the sensing of danger signals induces the expression of proinflammatory genes that, in turn, induce immune cell infiltration into the affected tissue to restore homeostasis.

### 3.4. Genes Influencing Host-Microbiome Relationship during Inflammation Have Altered Expression in IBD Patients

The relationship of the intestine and the microbiome in the lumen of the bowel is tightly regulated. In the healthy colon, the double mucin layer precludes the direct contact of bacteria and epithelium and protects the surface against hazardous agents [21]. The main component of this viscoelastic gel is mucin 2 (MUC2), which arranges the firmly adherent inner layer almost without bacteria, and the outer layer, which contains the microbiome. O-glycosylation of MUC2 serves as connection site and nutrient source for commensal flora [22,23]. The formation of glycan chains of glycoproteins is performed by glycosyltransferases. Glycosylation pathways are extremely complex with a contribution of several enzymes [21]. The altered glycan profile of MUC2 was observed in active UC patients compared to the normal glycosylation of control and inactive UC patients [24]. Arike et al. determined that the main glycosyltransferases assist in the O-glycosylation of MUC2 in mice colon. They highlighted the function of GalNAc-Ts, C1galt1, C1galt1c1, B3gnt6, Gcnt3 and Fut2 in the initiation step of O-glycan formation, while in the elongation stage, B4galnt2, B3gnt3, B3gnt7, B4galt1, B3galt5 and Chst1 have a crucial role [25]. The expression of seven out of 10 examined genes significantly decreased in the inflamed regions of the TNBS treated rat colons (data not shown). This may indicate the insufficient glycosylation of mucin in the inflamed regions of rat colons.

**Figure 2:**
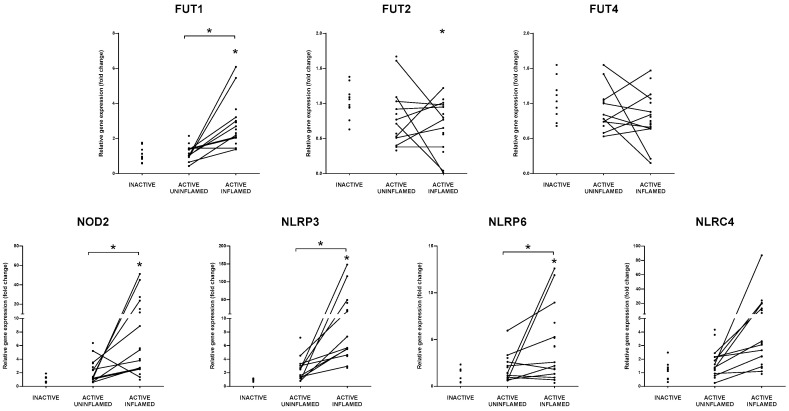
Distinct expression of genes regulating host-microbe interactions in IBD patients. The relative gene expression of FUTs: FUT1, FUT2, FUT4 and NLR receptors: NOD2, NLRP3, NLRP6, NLRC4 is shown from inactive (left), active uninflamed (middle) and active inflamed (right) colon samples of IBD patients. Dots represent individual values, and the lines connect uninflamed and inflamed samples from the same IBD patient; * *p* < 0.05.

The glycosylation in IBD does not only affect mucins, but also the formation of apical surface of intestinal epithelial cells (IECs) and immune cell tethering [26]. In addition to the fucosylation of MUC2 glycans, FUT2 also plays a role in the construction of glycocalyx of IECs. Together with FUT1, FUT2 synthetize H-antigen, defining the secretor status of ABO blood group antigens [27]. FUT1 is expressed on erythrocytes, vascular endothelium; FUT2 is present on epithelial cells and in body fluid [27]. IECs express Fut2 in response to the commensal microbiome, and the lack of Fut2 in mice leads to higher susceptibility to intestinal inflammation against pathogens [28]. Furthermore, genome-wide association studies demonstrate that the polymorphism of FUT2 is correlated with CD [29]. After TNBS treatment, expression of both Fut1 and Fut2 was significantly decreased in the inflamed regions compared to the uninflamed segments of rat colons (Supplementary Figure S3). In the case of IBD patients the expression of FUT1 increased while the level of FUT2 mRNA decreased in the active-inflamed colon samples (Figure 2), indicating some divergence between the course of IBD in humans and the irritant-induced colitis in rats.

Downregulation of FUT2 may lead to diminished IEC fucosylation and decreased protection against bacteria in the colon lumen, thereby promoting the progression of chronic inflammation.

### 3.5. Balancing between Danger and Tolerance

Perturbation of the commensal flora or the disintegration of glycosylated mucus barrier results in the direct contact of IECs and microbiome. The innate immunity, as a first line of defense, monitors environmental or host-derived factors and distinguishes between dangerous and safe signals. Pattern recognition receptors (PRRs) serve as sensors of pathogen associated molecular patterns (PAMPs) from microbes in the colon lumen or damage/danger associated molecular patterns (DAMPs) derived from dysfunctional cells of colonic tissue. NOD-like receptors (NLRs) belong to PRRs, whose significant activation were observed in the TNBS treated rat colons (Supplementary Tables S1 and S2, Figure S2).

Cytosolic NLRs are classified by their NATCH nucleotide-binding domains and leucine-rich repeats, and it is also the basis of their standard nomenclature [30]. NLRs act as regulators of antimicrobial response, colonic microbial ecology and mitochondrial antiviral immunity [31,32,33]. Moreover, they are the main components of the inflammasomes: a multiprotein complex that regulates the activation of proinflammatory cytokines in a caspase-1 dependent manner [34]. It is also important to note that the firstly identified CD susceptibility mutation affects the NLR family member NOD2, which recognizes the bacterial cell wall component peptidoglycan and induces the expression of proinflammatory genes [35,36]. TNBS treatment strongly influenced the expression of intracellular NOD like receptors (Supplementary Figure S2). Three members of the NLR family had increased expression in the inflamed colon regions (Nod2, Nlrp3, Nlrp12); in contrast, the levels of Naip5, Naip6, Nlrc4 and Nlrp6 mRNAs decreased (Supplementary Figure S2). Inflammasome complex forming NLRP3, NLRC4 and NLRP6 have a role in the maturation of interleukin-1 family cytokines [34,37]. NLRP3 is mostly expressed in myeloid cells, while the main sources of NLRP6 are epithelial cells of the intestine [38]. According to a two-step model, the production and activation of NLRP3 is induced by host and microbiota derived molecules, which results in the release of mature pro-inflammatory interleukins [38,39]. In line with the TNBS treated rat model, expression of NLRP3 also increased in the inflamed colon samples of IBD patients (Figure 2). In contrast, the expression of NLRP6 is regulated by host-colonizing bacteria and its activation eventuates IL-18 maturation, which in turn controls the production of antimicrobial peptides, hence promoting proper microbial composition [31,38]. In addition, NLRP6 is a negative regulator of the inflammatory response and prevents DSS-induced colitis in mice [31,40]. While in the rat model, expression of NLRP6 mRNA was slightly but not significantly elevated in the TNBS treated uninflamed and decreased in the inflamed colon regions, it was increased in the inflamed tissue samples from IBD patients (Figure 2, Supplementary Figure S2). The colonic expression of NLRC4 (also known as IPAF) that recognizes bacterial flagellin and type III secretion apparatus [41] was downregulated after TNBS treatment in the inflamed tissue areas, while in human colonoscopy samples, the mRNA level of NLRC4 increased (Figure 2, Supplementary Figure S2). Taken together, the expression pattern of the family of NOD-like receptors at the site of inflammation was characterized by the increased expression of inflammation promoting NLRs and decreased expression of NLRs with anti-inflammatory potential. 

### 3.6. Burst of Cytokine and Chemokine Expression

Ligand binding of PRRs induces the expression of chemotactic molecules, such as cytokines and chemokines from resident cells, resulting in the infiltration of immune cells to the damaged tissues [42].

**Figure 3:**
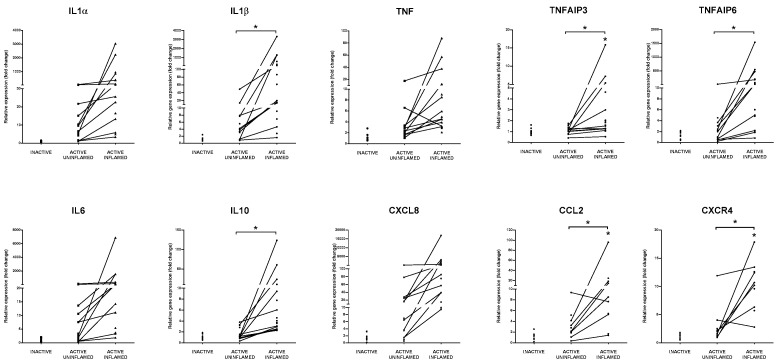
Global upregulation of proinflammatory cytokines and anti-inflammatory molecules indicate the presence of inflammatory response. The relative gene expression of IL1α, IL1β, TNF, TNFAIP3, TNFAIP6, IL6, IL10, CXCL8, CCL2 and CXCR4 cytokines, chemokines and anti-inflammatory genes is shown from inactive (left), active uninflamed (middle) and active inflamed (right) colon samples of IBD patients. Dots represent individual values, and the lines connect samples from the same IBD patient; * *p* < 0.05.

Members of the IL-1 family, such as IL-1α, IL-1β and IL-18 have an important role in the regulation of tissue homeostasis, inflammatory response and immune cell activation [43,44]. Although IL-1α and IL-1β use the same IL-1R1 for binding, their expression pattern and function differ [43]. Healthy barrier cells (such as endothelial and epithelial cells) constitutively produce considerable amounts of IL-1α, while in hematopoietic cells, its expression is induced by pro-inflammatory stimuli [45]. In contrast, the unique source of IL-1β are myeloid cells, where its inactive precursor must be cleaved by the inflammasome in a caspase-1 dependent manner to gain functional form [43]. According to the ‘inflammatory loop’ model, IEC-derived IL-1α initiates inflammation and recruitment of hematopoietic cells, which react to the inflammatory circumstances with further production of IL-1α, IL-1β and other pro-inflammatory mediators [45]. Bersudsky et al. examined the effect of neutralizing antibodies against IL-1α, IL-1β and IL-1 receptor in DSS-induced colitis model and observed mitigation of inflammation after inhibition of IL-1α, whereas IL-1β blocking exacerbated the symptoms [46]. Similarly to IL-1β, IL-18 maturation is related to inflammasomes; moreover, NLRP6 inflammasome deficiency causes inadequate IL-18 production [44]. IL-18 plays a role in the activation of NK and Th1 cells, thereby, it is involved in tumor control; however, the overproduction of IL-18 enhances intestinal permeability [43]. In the rat model of colitis, expression of both IL-18 and NLRP6 decreased (Supplementary Figures S2 and S5); in contrast, the expression of other members of IL-1 family were all induced, thus, the mRNA levels of IL-1α and IL-1β increased both in the human and rat tissues (Figure 3, Supplementary Figures S4 and S5).

Abnormal overexpression of pro-inflammatory TNF plays a role in the development of inflammatory diseases, such as IBD or psoriasis. As a pleiotropic cytokine, under homeostasis, it contributes to tissue regeneration, tumor control and defense against infections. However, uncontrolled TNF expression triggers inflammation, activation of endothelial cells, recruitment and survival of inflammatory cells and tissue destruction [47]. Both in the TNBS treated rat model of colitis and in IBD patients, the expression of TNF increased in the inflamed colon samples (Figure 3, Supplementary Figure S4).

TNF exerts its function through TNFRI and TNFRII (tumor necrosis factor receptor I and II) signaling that activates NF-κB pathway, thereby regulating the transcription of both pro- and anti-inflammatory molecules. Important negative regulators of inflammation are TNFAIP3 (TNF alpha induced protein 3, also known as A20) and TNFAIP6 (TNF alpha induced protein 6, also known as TSG6) [48,49]. Ubiquitin-editing enzyme TNFAIP3 is able to abolish NF-κB activation by removing ubiquitin chains from key components of TNFR signaling pathway (such as RIP1 and NEMO) that leads to their proteasomal degradation; notably, SNPs in TNFAIP3 are associated with CD [49]. TNFAIP6 belongs to the hyaluronan-binding protein family and exerts its anti-inflammatory effect by the binding to several chemokines (e.g., CCL2, CCL7, CXCL8). As a result, the chemokines are not able to interact with their binding partners, such as heparin or type I collagen [48]. The expression of all these negative regulators of inflammation increased in the inflamed colon regions of rat model of colitis as well as IBD patients, which correlates with the upregulation of their inducer TNF (Figure 3, Supplementary Figure S4). 

Members of the IL-6 cytokine family include IL-6, IL-11, oncostatin M (OSM), leukemia inhibitory factor (LIF) and cardiotropin-1 (CTF1) [50,51]. IL-1β and TNF are able to induce the transcription of IL-6 in both stromal and immune cells, thereby—under inflammatory conditions—IL-6 is a prominent predictor of disease activity. IL-6 is a key regulator of innate immune responses by controlling neutrophil accumulation, expression of chemokines and adhesion molecules [51]. Recently, high OSM levels in IBD patients were found to be associated with failure to respond to anti-TNF therapy [52]. We observed the significant upregulation of IL-6 and OSM signaling in the inflamed rat colons compared to the control and to the TNBS treated uninflamed tissue samples (Supplementary Tables S1 and S2) in line with the increased expression of IL-6 mRNA both in the TNBS induced model of colitis and in the colon biopsies of IBD patients (Figure 3, Supplementary Figure S4).

Activation of canonical IL-10 signaling pathway was also observed in the inflamed colon of TNBS treated rats (Supplementary Table S1). As an anti-inflammatory cytokine, IL-10 plays a role in tissue regeneration, and IL-10 polymorphisms are also associated with IBD [53]. We measured significantly elevated amount of IL-10 mRNA in the inflamed colons of TNBS treated rats and also in IBD patients (Figure 3, Supplementary Figure S4). 

In addition to the anti- and pro-inflammatory cytokines, chemokines are important regulators of inflammations by influencing leukocyte recruitment [54]. These small, secreted proteins bind to transmembrane chemokine receptors; hence, they activate intracellular pathways related to cell movement, such as cell arrest or migration [54]. CXCL8 (also known as IL-8, functional murine homologue of Cxcl1) exerts its effect through CXCR1 and CXCR2 receptors [55,56]. CXCL8/Cxcl1 plays a key role in the activation and recruitment of immune cells, such as neutrophils, monocytes and lymphocytes, while a blockade of CXCR2 or absence of Cxcl1 mitigate their infiltration to inflamed intestine [57]. We determined that in the inflamed colon of IBD patients, the mRNA level of CXCL8 significantly increased, just as its rodent homologue Cxcl1 and its receptor, Cxcr2 in rat samples (Figure 3, Supplementary Figures S4 and S5). CCL2 (also known as MCP-1) is responsible for the activation of monocytes and macrophages, while CCL2 deficiency in experimental colitis reduces severity of inflammation [58]. CXCR4 regulates the transmigration of T cells in the inflamed intestine [59]. We observed significant upregulation of both CCL2 and CXCR4 expression in the inflamed tissues in both rat and human samples (Figure 3, Supplementary Figure S5).

Novel therapeutic strategies are based on the suppression of pro-inflammatory cytokines or enhancement of anti-inflammatory mediators that targets a certain molecule. Many of the abovementioned molecules are already therapeutic targets for the reduction of IBD symptoms [60]. Currently, anti-TNF administration is the most widely used treatment against IBD symptoms. Although anti-TNF therapy seems to be effective in many cases, activation of alternative inflammatory pathways (e.g., IL-6 signaling) causes the development of a secondary non-response that turns anti-TNF therapy inadequate for more than 40% of IBD patients [60]. Similarly, although the blockade of IL-6 alleviates inflammatory symptoms, it in turn causes perforation of the intestine [60]. Interestingly, treatment with an IL-1 receptor agonist was ineffective in clinical trials, probably due to the different functions of its ligands [60]. IL-10, TNFAIP3 and TNFAIP6 are relatively novel targets for therapeutic applications. Due to their immunosuppressive effect, they have a potential to alleviate inflammatory symptoms of IBD patients. In line with this, Sala et al. successfully reduced intestinal inflammation in mice by TNFAIP6 administration [61]. Even though the systemic application of recombinant IL-10 has not achieved remission in IBD, the local production of IL-10 by IL-10-secreting *Lactobacillus lactis* is a promising approach that is in phase I/II clinical stage [53,60].

Even though the above examples represent a great leap forward in the treatment of IBD, targeting a certain molecule is not always fully successful. In line with this, our transcriptome analysis revealed the complex interplay of thousands of molecules, including cytokines and chemokines that are able to corporate, regulate or even replace each other’s function. Our genome wide transcriptome analysis revealed that upon inflammation many molecules belonging to a given class of effectors have synchronous alteration in their expression profile (such as upregulation of pro-inflammatory cytokines, etc.). From this perspective, targeting common regulator/s—such as microRNAs—of inflammation-associated molecules may have the potential to moderate the symptoms in most IBD patients more generally. 

Taken together, our data on the expression profile of cytokines and chemokines in both in vivo rat model of experimental colitis and inflamed colon samples of IBD patients are in line with numerous reports [60] highlighting the key role these molecules play in promoting the evolvement of the chronic inflammatory milieu in the bowel.

### 3.7. Support of Immune Cell Trafficking

**Figure 4:**
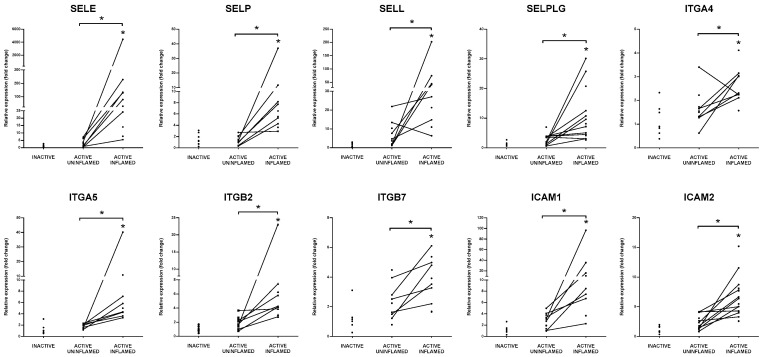
Elevated expression of integrins and selectins indicate increased adhesion of immune cells in the inflamed colon tissue. The relative gene expressions of selectins SELE, SELP, SELL and their ligand SELPLG, integrins ITGA4, ITGA5, ITGB2, ITGB7 and adhesion molecules ICAM1 and ICAM2 are shown from inactive (left), active uninflamed (middle) and active inflamed (right) colon samples of IBD patients. Dots represent individual values, and the lines connect uninflamed and inflamed samples from the same IBD patient; * *p* < 0.05.

Secretion of cytokines and chemokines promotes the activation of endothelial cells that, in turn, facilitate the transmigration of leukocytes. After inflammatory stimuli, endothelial cells express E-selectin (SELE) and p-selectin (SELP), while ligands, L-selectin (SELL) and SELPLG (also known as p-selectin glycoprotein ligand (PSGL1)) are located on the surface of activated leukocytes [62]. Their interaction is necessary for the capture of immune cells from the blood flow [63]. We detected a significant increase in the expression of all these molecules in the rat model of colitis (Supplementary Figure S5) and subsequently validated their expression in inflamed colons of IBD patients, corroborating significant induction of selectins (Figure 4).

**Figure 5:**
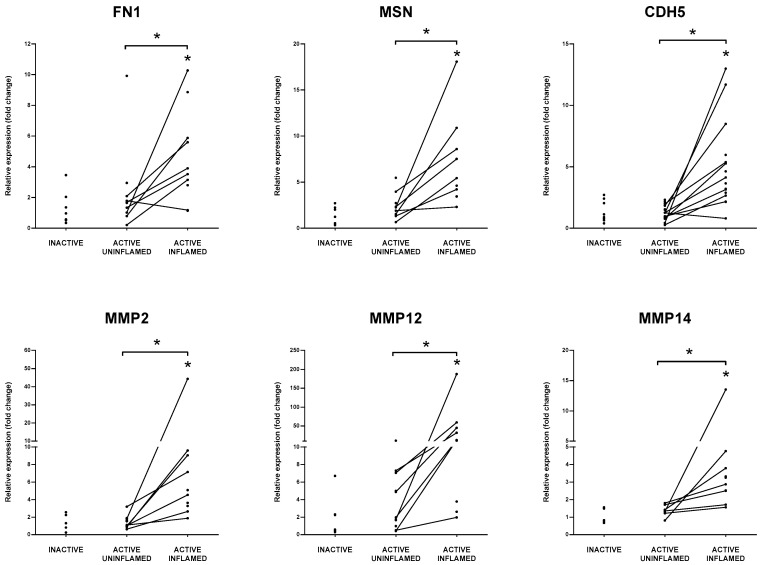
Collective upregulation of matrix remodelers and adhesion molecules facilitate migration of leukocytes through the endothelial cells of blood vessels. The relative gene expression of extracellular matrix component FN1, ERM family member MSN, cell adhesion molecules CDH5, matrix metalloproteinases MMP2, MMP12 and MMP14 is shown from inactive (left), active uninflamed (middle) and active inflamed (right) colon samples of IBD patients. Dots represent individual values, and the lines connect uninflamed and inflamed samples from the same IBD patient; * *p* < 0.05.

Chemoattractant-triggered leukocytes express integrin subunits (such as ITGA4, ITGA5, ITGB2, ITGB7), which assemble into ligands of ICAM1 and VCAM1 on the surface of endothelial cells promoting their rolling and arrest, while fibronectin (FN1) enhances this tight adherence by complex formation with integrins [63,64]. Moesin (MSN) interconnects ICAM1 to cytoskeleton components providing structural support during migration [62]. Similar to selectins, the expression of the above mentioned adhesion molecules was all upregulated after TNBS treatment of rats (Supplementary Figure S5) as well as in the inflamed colons of IBD patients (Figure 4 and Figure 5).

Rolling of leukocytes is necessary for finding the exit site from the blood vessel that is supported by the endothelial ligand ICAM2 [63]. Docking of circulating immune cells onto the endothelial surfaces induce the production of matrix metalloproteinases, including MMP2, MMP12 and MMP14. MMPs rearrange ECM and degrade junctional molecules, such as CDH5 (VE-cadherin, vascular endothelial cadherin) or claudins to open the interendothelial way for transmigrating leukocytes [65]. MMPs also have crucial role in the processing of cytokines and chemokines, such as TNF, IL-1β and CXCL1 [66,67,68]. In the inflamed rat and human colon samples, we observed a synchronous upregulation of ICAM2, MMP2, MMP12, MMP14 and CDH5 (Figure 5 and Supplementary Figure S5) that may promote the diapedesis of immune cells into the involved tissue/s.

The fucosyltransferases mentioned earlier are responsible for the synthesis of CD15 (Lewis-related carbohydrate Le^x^) antigens [27], in addition to controlling glycolysation of IECs and mucin glycoproteins. FUT4 and FUT9 take a part in the formation of CD15 which is a cell surface marker of mature neutrophils, monocytes and promyelocytes. Here, CD15 as a carbohydrate ligand of selectins, contributes to leukocyte adhesion and diapedesis [69,70]. Cancer cells express high level of sialyl-Le^x^, and as a cancer marker, elevated level of sialyl-Le^x^ is associated with high chance of metastasis [71]. Through the activation of the PI3K/Akt and NF-κB pathways, FUT4 regulates the expression of EMT transcription factor SNAIL and MMP9, thus promoting EMT [72]. Moreover, it is also upregulated in human colorectal carcinomas, and the knockdown of FUT4 decreases the mesenchymal characteristic in breast cancer cell lines [72,73]. In the TNBS-induced experimental model of colitis, we detected elevated expression of Fut4 and Fut9 in the TNBS-treated uninflamed tissues, and decreased expression in the inflamed regions, respectively (Supplementary Figure S3). Yet, in the human samples we have not detected any significant alteration of FUT4 (Figure 2). Importantly, despite the reported expression of FUT9 in human colon [74], we were unable to detect measureable amount of FUT9 transcript in the colon samples independently of the status of inflammation, despite using three different primer and probe sets (data not shown).

Recruitment of immune cells is essential for the effective restoration of tissue homeostasis, which is promoted by the increased expression of adhesion molecules. Here we have shown data on the global activation of signaling pathways and increased expression of molecules associated with the migration and diapedesis of immune cells in the involved colons of both rats after colitis induction and IBD patients. Although the leukocytes may effectively eliminate the microbial agents inducing acute inflammation, their excessive and prolonged accumulation also facilitates the establishment of chronic inflammation. Blocking of adhesion molecules, such as integrins or their ligand ICAM1, is a promising therapeutic approach, for instance, anti-α4β7 treatment, which represses the intestinal homing of lymphocytes, is now used in the treatment of IBD patients [75,76].

### 3.8. Definition of Immune Cell Signature

Inhibition of leukocyte recruitment and infiltration to the inflamed tissue by blocking pro-inflammatory cytokines and adhesion molecules proved to be relevant in IBD treatment. The role of immune cells in IBD pathogenesis is indisputable, just as their dynamic fluctuation in the colon tissue. In contrast, the scale of their exact contribution in the inflammatory process is still debated. Cellular structure of colon tissue is characterized by resident and infiltrating cells. All of them have distinct or overlapping functions in the maintenance of mucosal tissue homeostasis. Under pathological conditions, such as IBD-associated intestinal inflammation, activation of innate and adaptive immune responses triggers significant accumulation of immune cells in the involved tissues. Inflammation subsequently induces structural reconstruction of colonic mesenchyme, for example colitis-specific fibroblast subsets affect barrier functions and boost inflammation [77].

Sequencing of the full transcriptome of colon tissue samples from in vivo TNBS-treated rat model of experimental colitis facilitates the monitoring of the global gene expression alteration of a heterogeneous cell subset that is currently present at the uninflamed or in the inflamed regions (Figure 1). Novel bioinformatics tools enable the quantitative estimation of proportion of different immune cell types in these complex tissue samples based on transcriptomic data, by using a network–based deconvolution approach [17]. Recently, defined markers whose collective observation determines gene signatures of seven distinct immune cell types, termed as ImSig, have been recently released [17]. Since this application is based on data from human tissue transcriptomes, first we had to identify the homologous *Rattus norvegicus* gene IDs of the *Homo sapiens* Immune Signature genes. Out of the 569 immune human signature genes, we have identified 476 IDs in the Complete List of Vertebrate Homology Classes database and additional 41 homolog/orthologue IDs were identified manually using the resources of NCBI and HGNC databases (Supplementary Tables S4 and S5). Next, we determined the number of overlapping genes between ImSig and our transcriptomic data and applied the recommended 75% as lower bound, while for feature selection, we used the correlation threshold at r = 0.7 (Supplementary Table S3). As a result, we excluded the *NK cells* and *Plasma cells* categories from further analysis, since the number of rat homologous have not reached the recommended threshold.

**Figure 6:**
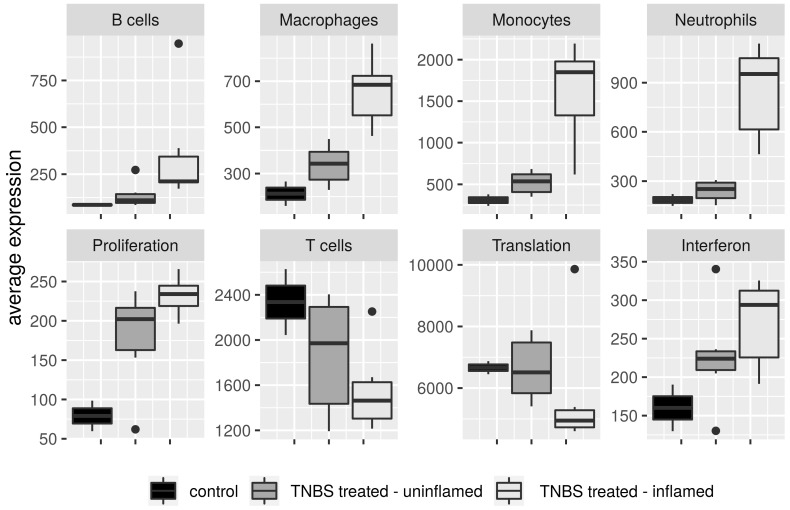
Immune cell composition of colon tissues from in vivo model of IBD. Average expression of immune signature genes of B cells, macrophages, monocytes, neutrophils and T cells, and pathway signature genes of Interferon, Proliferation and Translation categories between control, TNBS-treated uninflamed and TNBS-treated inflamed rat colon samples.

Based on coexpression of signature genes of ImSig approach, we observed the accumulation of macrophages, monocytes and neutrophils primarily in the TNBS-treated inflamed colon samples, while in the uninflamed regions their amount have not changed considerably compared to the control samples (Figure 6). Cells of the adaptive immune response, B cells and T cells have not changed as dramatically as macrophages, monocytes or neutrophils; furthermore, we detected higher deviation among biological replicates. Even so, the average expression of B-cell signature genes moderately increased in the inflamed samples; strikingly, T-cell gene signature diminished in the involved tissues (Figure 6).

This is, however, in line with a recent work reporting inverse rate of T cell subtypes in IBD patients compared with healthy controls, namely elevated percentage of CD4+ T cells, Tregs and T_CM_, while the amount of CD8+ T cells and CD103+ T cells decreased [78]. ImSig currently does not distinguish between T-cell subpopulations; therefore, we suspect that the overall reduction of T cells in the inflamed rat colons may be the consequence of the lower CD8+ and/or CD103+ T-cell numbers. 

Several studies highlighted the contribution of abnormal adaptive immune response in the pathogenesis of IBD; however, the cellular components of mucosal innate immunity gained attention only in the last few years [78,79,80]. Notably, the dysregulated transformation of infiltrating monocytes to pro-inflammatory cytokine producing cells, as well as altered macrophages with ability to induce T_H_17 differentiation in patients with IBD, suggest the relevance of innate immune cells as triggers of pathogenic intestinal inflammation [79,81]. On the other hand, the significant recruitment of neutrophils is well-described in IBD patients, but their role is contradictory, as some reports suggest their advantageous role during intestinal inflammation, while others described tissue damaging effects [82,83]. 

Besides immune signatures, ImSig determines pathway signatures such as *Interferon*, *Proliferation* and *Translation*. In our transcriptome dataset, despite the high overlap with ImSig *Interferon* signature genes (48/59) and *Translation* signature genes (61/84), the median correlation has not reached the required 0.7 correlation threshold value (Supplementary Table S3). *Proliferation* signature genes, however, reached the expected overlap and correlation threshold (85.71% and 0.77, respectively); hence, their average expression unambiguously increased after TNBS treatment. Interestingly, activation of this gene signature was detectable not just in the inflamed but also in the TNBS-treated uninflamed colon samples (Figure 6). For the replacement of necrotic cells in the damaged tissues, the formation of novel cells is essential to restore homeostasis. Accordingly, alterations in the mRNA expression pattern of genes related to enhanced epithelial proliferation and differentiation in the TNBS-treated rat colons are expectable, together with the genomic evidences of the resolution of the inflammation.

## 4. Conclusions

Animal experiments are still widely used when studying various aspect of human diseases and treatment options, with ~90% of laboratory animal experiments conducted in mice and rats. Despite many advantages of these models, there are undoubted limitations, some arising from differences between species others due to differences between diseases. Here, we used a rat model of TNBS-induced experimental colitis where inflammation develops in 72 h, in contrast, IBD is a chronic disease which often has very long duration. Even though some of the data generated on rat model could not be validated on human samples (e.g., expression profile of FUT1, NLRP6 and NLRC4), the majority (30 out of 33) of the gene expression changes were identical, demonstrating that animal models—in this case TNBS-induced colitis—are still useful tools in deciphering molecular mechanisms underlying human diseases, such as IBD.

In the life of IBD patients inactive and active disease phases fluctuate, while the localization of inflamed and uninflamed colon regions alternates along the digestive tract. This course is coupled with the fluctuation of the expression of genes regulating inflammation. Here, we report a genome-wide alteration in the expression pattern of inflammation-related gene sets in the TNBS-treated rat model of colitis, and the validation of a subset of these genes on samples derived from IBD patients. As a barrier tissue, the intestinal epithelium is constantly exposed to the colonizing bacteria from the lumen; in line with the perturbation of commensal gut bacteria, termed as dysbiosis, that is characteristic in IBD patients. Here, we measured the expression pattern of genes related to glycosylation and recognition of PAMPs/DAMPs. Absence of fucoslytransferases (FUT2) may affect the protection of colonic epithelium, while damaged cell-derived alarmins activate various immune cells through pattern recognition receptors, such as NLRs. Induction of inflammatory cytokines and chemokines leads to the recruitment and homing of leukocytes.

Colon tissue is composed of several cell types, and all of them contribute differently to the maintenance of mucosal homeostasis or to the regulation of immune response. In the present work, we monitored the actual gene expression alteration of a heterogenic cell population present in the uninflamed or inflamed regions of rat and human tissue samples. The rapid development of single-cell sequencing methods allows for increased resolution of our transcriptome analysis by applying single-cell RNA-Seq (scRNA-Seq) and determine the gene expression alteration of specific cell types in the inflamed vs. uninflamed colon tissues.

## Figures and Tables

**Table 1:** Demographic data for study participants. Patient records were reviewed for disease state (inactive, active), tissue state (inflamed, uninflamed), Inflammatory Bowel Disease (IBD) type (Crohn’s Disease, CD; Ulcerative Colitis, UC; IBD indeterminate, UC/CD), biopsy location (colon ascendens/transversum/descendens/sigmoideum), age (year, y), sex (male, M; female, F) and disease duration (year, y).

Disease State	IBD	Patient ID	Sample ID	Sex	Age, y	Biopsy Location	Tissue State	Disease Duration, y
inactive	CD	1	H1	M	47	colon descendens	−	27
inactive	CD	2	H3	M	32	colon ascendens	−	7
inactive	UC	3	H5	F	36	colon ascendens	−	16
inactive	CD	9	H27	F	39	colon ascendens	−	17
inactive	CD	9	H28	F	39	colon descendens	−	17
inactive	UC	16	H50	F	72	colon ascendens	−	12
inactive	UC	16	H51	F	72	colon descendens	−	12
inactive	UC	33	H99	M	56	colon ascendens	−	21
inactive	UC	33	H100	M	56	colon transversum	−	21
inactive	UC	33	H101	M	56	colon descendens	−	21
active	UC/CD	4	H10	F	41	colon ascendens	uninflamed	20
active	UC/CD	4	H12	F	41	colon transversum	uninflamed	20
active	UC	6	H18	F	27	colon ascendens	uninflamed	2
active	UC	6	H21	F	27	colon descendens	uninflamed	2
active	CD	10	H31	F	43	colon transversum	uninflamed	9
active	UC	11	H32	F	44	colon ascendens	uninflamed	11
active	CD	12	H35	M	37	colon ascendens	uninflamed	3
active	CD	15	H48	F	37	colon ascendens	uninflamed	12
active	CD	15	H49	F	37	colon descendens	uninflamed	12
active	UC	17	H56	M	51	colon descendens	uninflamed	14
active	UC	18	H58	F	47	colon sigmoideum	uninflamed	17
active	UC	20	H62	F	41	colon sigmoideum	uninflamed	12
active	CD	22	H68	F	24	colon transversum	uninflamed	6
active	UC	28	H86	M	49	colon descendens	uninflamed	20
active	CD	29	H88	M	33	colon ascendens	uninflamed	11
active	UC/CD	4	H9	F	41	colon ascendens	inflamed	20
active	UC/CD	4	H11	F	41	colon transversum	inflamed	20
active	UC	6	H19	F	27	colon ascendens	inflamed	2
active	UC	6	H20	F	27	colon descendens	inflamed	2
active	CD	8	H25	M	30	colon transversum	inflamed	14
active	CD	12	H36	M	37	colon descendens	inflamed	3
active	UC	13	H39	F	33	colon descendens	inflamed	11
active	UC	17	H54	M	51	colon ascendens	inflamed	14
active	UC	17	H55	M	51	colon transversum	inflamed	14
active	UC	18	H57	F	47	colon sigmoideum	inflamed	17
active	UC	20	H63	F	41	colon sigmoideum	inflamed	12
active	CD	22	H69	F	24	colon sigmoideum	inflamed	6
active	UC	24	H74	M	42	colon transversum	inflamed	21
active	UC	24	H75	M	42	colon sigmoideum	inflamed	21
active	UC	28	H87	M	49	colon sigmoideum	inflamed	20
active	CD	29	H89	M	33	colon sigmoideum	inflamed	11

**Table 2:** SybrGreen primer sets used in QPCR experiments for rat samples.

Gene ID	Forward (5′-3′)	Reverse (5′-3′)
**Cxcl1**	CATTAATATTTAACGATGTGGATGCGTTTCA	GCCTACCATCTTTAAACTGCACAAT
**Fut1**	AGGACCCGTTTCTCAAGCTG	CTATCCGGAGCCCACTCAAC
**Fut2**	ACTTCCACCATCATCCACCTC	CTCTGGGCTTTCTGTGTTTCC
**Fut4**	AAGCTACAGCATGAGAGCCG	AGAGGAGGTCCGGGGTAATC
**Fut9**	CAACAAATCCCATGCGGTCC	CAATGCCACTCTTTTGGGGG
**Il1β**	CAGGAAGGCAGTGTCACTCA	AGACAGCACGAGGCATTTTT
**Il6**	CACTTCACAAGTCGGAGGCT	TCTGACAGTGCATCATCGCT
**Nlrc4**	GCGAAACCTGAAGAAGATGC	AACGCTCAGCTTGACCAAAT
**Nlrp3**	GCTGCTCAGCTCTGACCTCT	AGGTGAGGCTGCAGTTGTCT
**Nlrp6**	TACCTGGTCATTGTGCTCCA	TCAGAGGCTGAGGATGTGTG
**Nod2**	TCCTTGCACACAAGCAGAAC	TGATCAGCCACAACTTCAGC
**Tnf**	ATGGGCTCCCTCTCATCAGT	GCTTGGTGGTTTGCTACGAC

**Table 3:** SybrGreen primer sets used in QPCR experiments for human samples.

Gene ID	Forward (5′-3′)	Reverse (5′-3′)
**CCL2**	CCTTCATTCCCCAAGGGCTC	CTTCTTTGGGACACTTGCTGC
**CDH5**	GAGAATGACAATGCCCCGGA	ACTGTGATGTTGGCCGTGTT
**CXCR4**	GGTGGTCTATGTTGGCGTCT	ACACAACCACCCACAAGTCA
**FN1**	GGGCGAGGGAGAATAAGCTG	TCTCAGCTATGGGCTTGCAG
**FUT1**	TGGCCGGTTTGGTAATCAGA	TCCGACATCCAGTCGTGAAG
**FUT2**	TGGACCTTCTACCACCACCT	CCACCCCCTTCCACACTTTT
**FUT4**	GCCTCGTACCTGCTTTTCCT	AGTTCCGTATGCTCTTGGGC
**FUT9**	CCATACCTACGGGCAAGCAT	ACAACAGGTACAGAGCCAGC
**ICAM1**	TGACCGTGAATGTGCTCTCC	TCCCTTTTTGGGCCTGTTGT
**ICAM2**	TCTGCTGTCCAGGATCGGA	TTTCCACTGAGCCTGTTCGT
**IL1β**	AGCTGGAGAGTGTAGATCCCAA	GGGAACTGGGCAGACTCAAA
**ITGA4**	CGCTTCAGTGATCAATCCCG	CTCCATTAGGGCTACCCAGC
**ITGA5**	TGGCCTTCGGTTTACAGTCC	GGAGAGCCGAAAGGAAACCA
**ITGB2**	TGGATGAGAGCCGAGAGTGT	CAGCAGGAGAATGCCGATCA
**ITGB7**	TCGCAGCACAGAGTTTGACT	CTGTACCACGTTGCTGGAGT
**MMP12**	CGGGCAACTGGACACATCTA	CCTCACGGTTCATGTCAGGT
**MMP14**	GGCGGGTGAGGAATAACCAA	ACGCCTCATCAAACACCCAA
**MMP2**	GCCCTGATGGCACCCATTTA	GTCAGGAGAGGCCCCATAGA
**MSN**	TCTGCAAGTGAAAGAGGGCA	CCTCCCACTGGTCCTTGTTG
**NLRC4**	GGACAATAGCCGAGCCCTTA	CCACCTTCTCGCAGCAAATG
**NLRP3**	AAGGCCGACACCTTGATATG	CCGAATGTTACAGCCAGGAT
**NLRP6**	CAGTTCTCAAGGCACCACAA	TCACTCAGCATACGCAGTCC
**NOD2**	CTCCATGGCTAAGCTCCTTG	CACACTGCCAATGTTGTTCC
**SELE**	GCCTGCAATGTGGTTGAGTG	GGTACACTGAAGGCTCTGGG
**SELL**	GCCTGCCACAAACTAAAGGC	TCACAAACTGACACTGGGGC
**SELP**	CCAACCTGCAAAGGCATAGC	GTGTGAATCCAGCGTTGCAG
**SELPLG**	AGAAGGAGATAAGATGGCTGGTG	ACTCATATTCGGTGGCCTGTC

**Table 4:** TaqMan Gene Expression Assays.

Gene ID	Assay Number
**IL1α**	Hs00174092_m1
**IL6**	Hs00174131_m1
**IL8 (CXCL8)**	Hs00174103_m1
**IL10**	Hs99999035_m1
**TNFAIP3**	Hs00234713_m1
**TNFAIP6**	Hs01113602_m1
**TNF**	Hs00174128_m1
**18S RNA**	Hs99999901_s1

## Supplementary Materials

The following are available online at https://www.mdpi.com/2218-273X/10/7/974/s1, Figure S1: Validation of RNA-Seq results by the comparison to our previous QPCR results related to epithelial-to-mesenchymal transition [13,15], Figure S2: Expression pattern of NOD-like receptors in experimentally induced colitis, Figure S3: Diverse expression pattern of FUTs at the site of colon inflammation in rat experimental colitis, Figure S4: Synchronous upregulation of inflammatory cytokines in the involved colon tissues, Figure S5: Canonical pathway of Agranulocyte adhesion and diapedesis in the TNBS induced rat model of IBD, Table S1: List of significantly activated canonical pathways in the in vivo rat model of IBD based on *p*-value, calculated by Ingenuity Pathway Analysis (IPA) application, Table S2: List of significantly activated canonical pathways in the in vivo rat model of IBD based on z-score, calculated by IPA application, Table S3: Summary of genes used for the determination of immune and pathway signatures of in vivo model of IBD by ImSig, Table S4: List of human and rat gene IDs used for gene signature analysis by ImSig (Part 1), Table S5: List of human and rat gene IDs used for gene signature analysis by ImSig (Part 2).
