# Supplementary material for: Transcriptome Based Profiling of the Immune Cell Gene Signature in Rat Experimental Colitis and Human IBD Tissue Samples
Source: Biomolecules. 2020 Jun 29;10(7):974. doi: 10.3390/biom10070974 (PMC7407160; doi:10.3390/biom10070974)
Supplement: Supplementary file 1 [file biomolecules-10-00974-s001.pdf]

Supplementary figures and tables

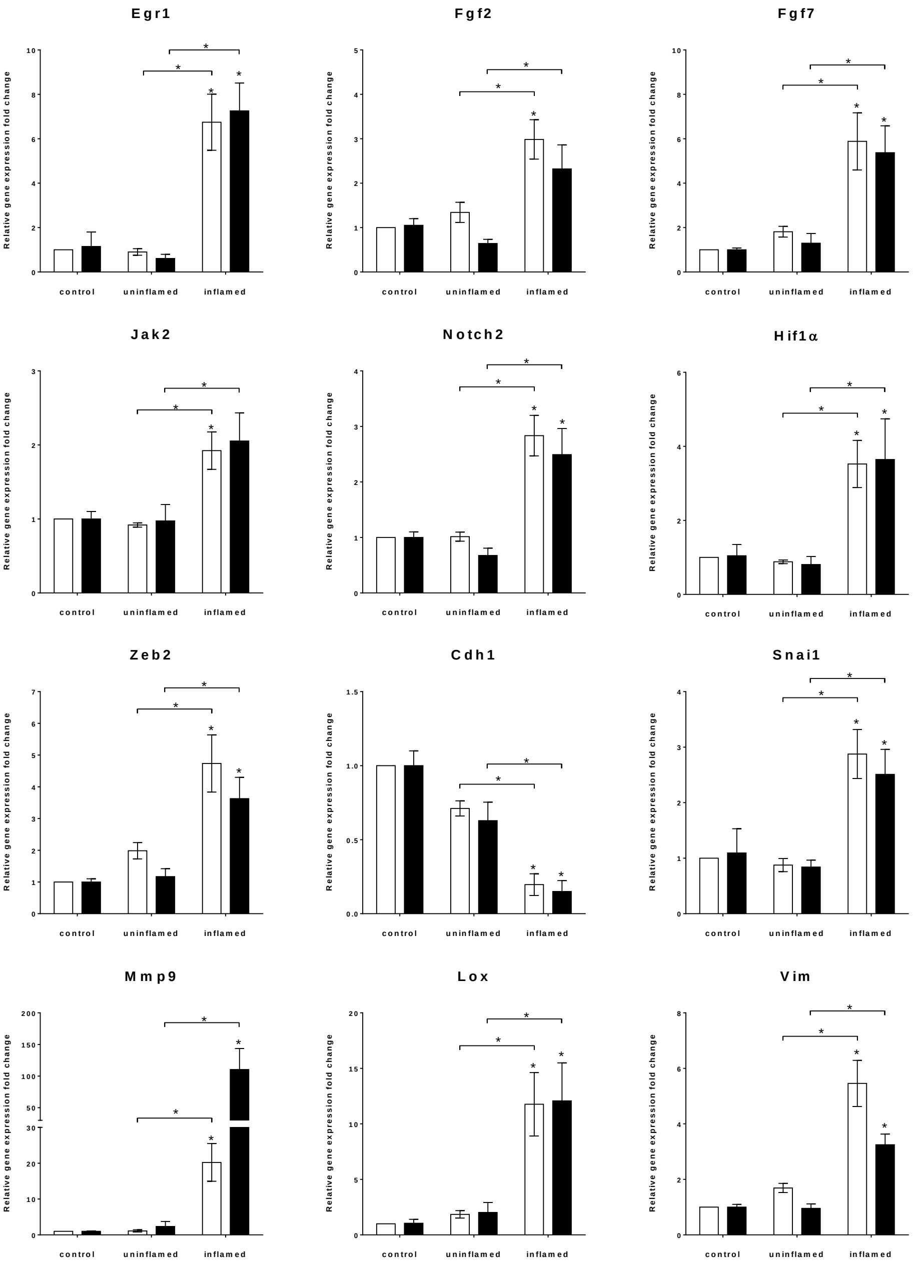

Supplementary Figure 1. Validation of RNA-Seq results by the comparison to our previous QPCR results related to epithelial-to-mesenchymal transition [13, 15]. The relative gene expression of Egr1, Fgf2, Fgf7, Jak2, Notch2, Hif1α, Zeb2, Cdh1, Snai1, Mmp9, Lox and Vim is shown from control (left columns), uninflamed (middle columns) and inflamed (right columns) rat colon sections. White and black columns represent RNA-Seq and previously published QPCR data, respectively. Data are presented as the mean ± SEM; *$p < 0.05$.

Supplementary table 1 List of significantly activated canonical pathways in the *in vivo* rat model of IBD based on *p*-value, calculated by Ingenuity Pathway Analysis (IPA) application. The abbreviations “UI—C,” “I—C” and “I—UI” represent the TNBS treated—uninflamed vs. control, TNBS-treated—inflamed vs. control and TNBS treated—inflamed vs. TNBS treated—uninflamed comparisons.

Ingenuity Canonical Pathways	UI - C -log(p-value)	I - C -log(p-value)	I - UI -log(p-value)
Granulocyte Adhesion and Diapedesis	N/A	18,00	19,00
Agranulocyte Adhesion and Diapedesis	N/A	13,40	16,00
Leukocyte Extravasation Signaling	N/A	11,80	8,01
Hepatic Fibrosis / Hepatic Stellate Cell Activation	N/A	11,30	13,70
Role of Macrophages, Fibroblasts and Endothelial Cells in Rheumatoid Arthritis	N/A	8,19	9,20
IL-10 Signaling	N/A	7,90	7,31
Atherosclerosis Signaling	N/A	7,84	11,60
phagosome formation	N/A	7,52	6,55
Role of Osteoblasts, Osteoclasts and Chondrocytes in Rheumatoid Arthritis	N/A	6,40	4,33
TREM1 Signaling	N/A	5,70	11,30
LPS/IL-1 Mediated Inhibition of RXR Function	1,74	5,62	10,60
Oxidative Phosphorylation	N/A	5,59	18,50
Hepatic Cholestasis	N/A	5,21	8,08
Acute Phase Response Signaling	N/A	5,17	6,13
LXR/RXR Activation	N/A	5,09	10,10
Mitochondrial Dysfunction	N/A	5,04	16,40
Retinoate Biosynthesis I	3,34	4,77	2,79
Inhibition of Matrix Metalloproteases	N/A	4,56	5,33
Dendritic Cell Maturation	N/A	3,85	4,31
Role of Pattern Recognition Receptors in Recognition of Bacteria and Viruses	N/A	3,82	5,19
NF-κB Signaling	1,35	3,81	4,27
Axonal Guidance Signaling	1,31	3,77	2,44
IL-6 Signaling	N/A	3,71	4,65
PPAR Signaling	N/A	3,51	4,50
Xenobiotic Metabolism Signaling	N/A	3,23	4,50
PPARα/RXRα Activation	1,31	3,20	3,29
Communication between Innate and Adaptive Immune Cells	N/A	2,88	5,27
Altered T Cell and B Cell Signaling in Rheumatoid Arthritis	N/A	2,67	5,02
Antioxidant Action of Vitamin C	N/A	2,48	6,20
FXR/RXR Activation	N/A	2,40	6,63
VDR/RXR Activation	N/A	2,11	4,91
Human Embryonic Stem Cell Pluripotency	1,63	1,56	1,37
STAT3 Pathway	1,36	N/A	1,71

Supplementary Table 2. List of significantly activated canonical pathways in the *in vivo* rat model of IBD based on z-score, calculated by IPA application. Abbreviations as "UI—C," "I—C" and "I—UI" represent the TNBS treated—uninflamed vs. control, TNBS-treated—inflamed vs. control and TNBS treated—inflamed vs. TNBS treated—uninflamed comparisons.

Ingenuity Canonical Pathways	UI-C z-score	I-C z-score	I-UI z-score
Acute Phase Response Signaling	NaN	4,02	4,27
TREM1 Signaling	NaN	3,55	3,77
IL-6 Signaling	NaN	2,75	3,78
Oncostatin M Signaling	NaN	2,65	2,83
Fcγ Receptor-mediated Phagocytosis in Macrophages and Monocytes	NaN	2,40	3,13
Role of Pattern Recognition Receptors in Recognition of Bacteria and Viruses	NaN	2,36	2,24
Interferon Signaling	NaN	2,24	1,41
PI3K/AKT Signaling	NaN	2,18	1,89
Leukocyte Extravasation Signaling	NaN	2,16	3,77
Colorectal Cancer Metastasis Signaling	NaN	2,08	3,48
Complement System	NaN	2,00	NaN
p38 MAPK Signaling	NaN	1,96	2,45
Production of Nitric Oxide and Reactive Oxygen Species in Macrophages	NaN	1,92	3,09
GM-CSF Signaling	NaN	1,90	2,31
Pancreatic Adenocarcinoma Signaling	NaN	1,60	2,50
Role of NFAT in Regulation of the Immune Response	NaN	1,53	2,86
HMGB1 Signaling	NaN	1,40	3,41
IL-8 Signaling	NaN	1,22	3,18
Rac Signaling	NaN	1,07	2,71
VEGF Signaling	NaN	1,00	2,71
eNOS Signaling	NaN	0,85	2,84
Role of NANOG in Mammalian Embryonic Stem Cell Pluripotency	NaN	0,82	2,00
Tec Kinase Signaling	NaN	0,78	2,35
PKCθ Signaling in T Lymphocytes	NaN	0,73	2,32
PDGF Signaling	NaN	0,58	2,71
iCOS-iCOSL Signaling in T Helper Cells	NaN	0,30	2,31
p53 Signaling	NaN	-0,28	-2,33
PPARα/RXRα Activation	NaN	-1,15	-2,13
Tumoricidal Function of Hepatic Natural Killer Cells	NaN	-2,00	NaN
Gαq Signaling	NaN	-2,04	0,43
Glioblastoma Multiforme Signaling	NaN	-2,07	0,50
Neuregulin Signaling	NaN	-2,12	-0,30
CXCR4 Signaling	NaN	-2,13	0,24
Melatonin Signaling	NaN	-2,33	NaN
Neuropathic Pain Signaling In Dorsal Horn Neurons	-2,00	-2,50	NaN
Sperm Motility	NaN	-2,52	-0,23
LXR/RXR Activation	NaN	-2,75	-2,67
PPAR Signaling	NaN	-2,99	-4,08
Thrombin Signaling	NaN	-3,27	-0,82

Nod - like receptors

Gene ID	Ciita	Naip5	Naip6	Nod1	Nod2	Nlrc3	Nlrc4	Nlrc5	Nlrp1a	Nlrp1b	Nlrp3
UI - C	0,9	-1,5	-0,7	0,3	-0,1	0,8	-0,1	0,2	-0,1	-0,6	0,5
I - C	1,6	-2,4	-1,5	0,6	2,7	1,7	-1,0	0,8	0,2	-0,9	3,6
I - UI	0,7	-0,9	-0,8	0,3	2,8	0,8	-0,9	0,6	0,4	-0,4	3,1

Gene ID	Nlrp4	Nlrp4a	Nlrp4b	Nlrp4f	Nlrp5	Nlrp6	Nlrp9	Nlrp10	Nlrp12	Nlrp14	Nlrx1
UI - C	-0,3	-0,1	0,6	0,1	0,2	1,0	0,0	0,4	-0,3	0,2	0,0
I - C	-0,1	0,3	0,9	0,0	1,0	-3,0	0,1	0,4	0,9	1,1	0,0
I - UI	0,2	0,4	0,3	-0,2	0,8	-4,0	0,1	0,1	1,2	0,8	0,0

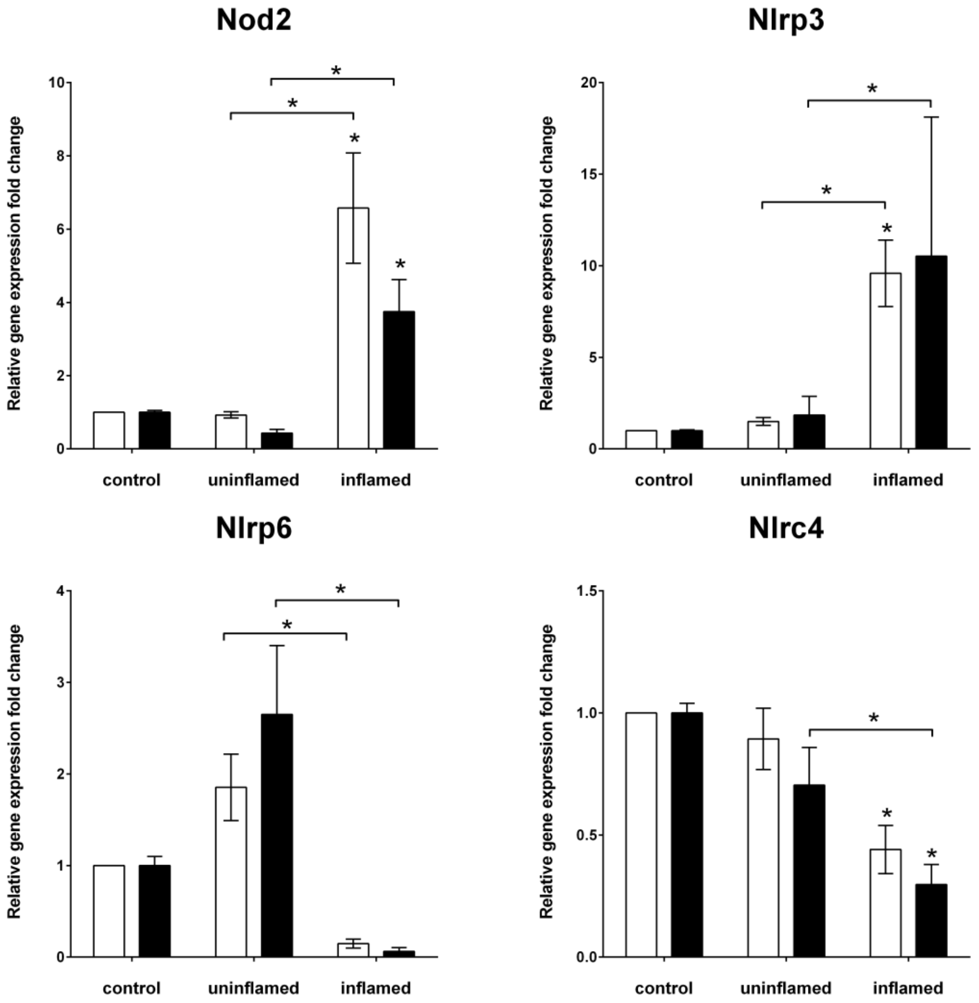

Supplementary Figure 2. Expression pattern of NOD-like receptors in experimentally induced colitis. Heat map shows gene expression alteration of NLR receptors between control, TNBS-treated uninflamed and TNBS-treated inflamed rat colon samples, where red marks elevation, while blue marks decrease by at least two-fold in expression level; false discovery rate (FDR) < 0.05. Bar plots show the expression alteration of Nod2, Nlrp3, Nlrp6 and Nlrc4 between rat sample groups; white and black columns represent RNA-Seq and QPCR data, respectively, $*p < 0.05$. Abbreviations as “UI—C,” “I—C” and “I—UI” represent the TNBS treated—uninflamed vs. control, TNBS-treated—inflamed vs. control and TNBS treated—inflamed vs. TNBS treated—uninflamed comparisons.

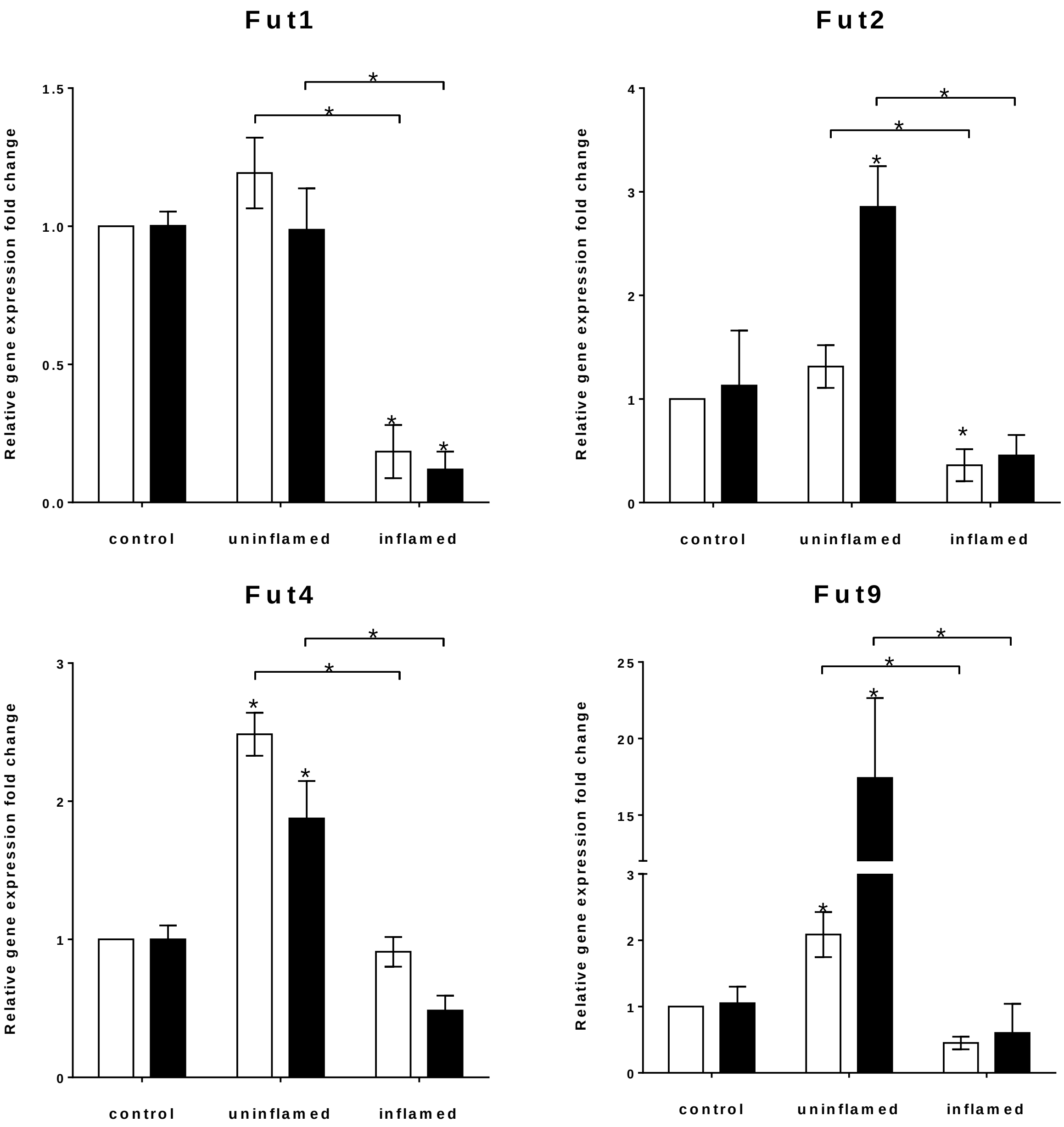

Supplementary Figure 3. Diverse expression pattern of FUTs at the site of colon inflammation in rat experimental colitis**.** The relative gene expression of Fut1, Fut2, Fut4 and Fut9 is shown from control (left columns), uninflamed (middle columns) and inflamed (right columns) rat colon sections. White and black columns represent RNA-Seq and QPCR data, respectively. Data are presented as the mean ± SEM; *$p < 0.05$.

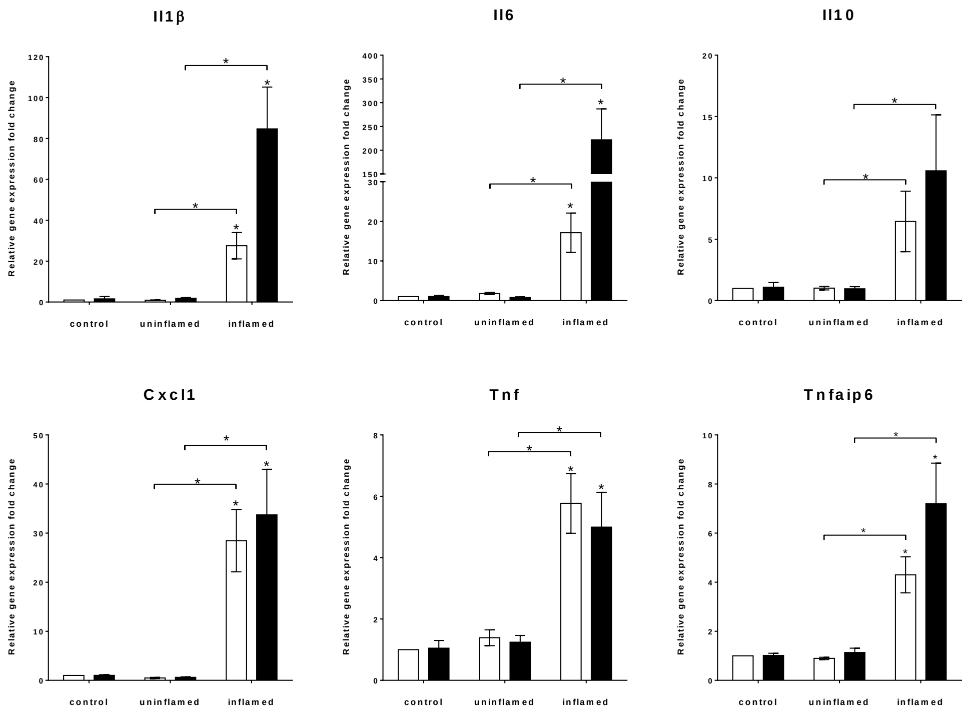

Supplementary Figure 4. Synchronous upregulation of inflammatory cytokines in the involved colon tissues. The relative gene expression of Il1β, Il6, Il10, Cxcl1, Tnf and Tnfaip6 is shown from control (left columns), uninflamed (middle columns) and inflamed (right columns) rat colon sections. White and black columns represent RNA-Seq and QPCR data, respectively. Data are presented as the mean ± SEM; *$p < 0.05$.

A.

Agranulocyte Adhesion and Diapedesis			
Gene ID	UI - C	I - C	I -UI
C5ar1	1,6	3,9	2,3
Ccl11	0,1	1,8	1,8
Ccl12	0,2	2,2	2,0
Ccl2	-0,8	3,2	4,0
Ccl28	-0,8	-2,5	-1,7
Ccl3	-0,7	6,0	6,8
Ccl4	0,8	3,6	2,8
Ccl7	-0,3	3,5	3,8
Cdh5	0,8	2,1	1,3
Cldn12	-0,3	-1,3	-1,0
Cldn23	-0,7	-3,4	-2,7
Cldn3	-0,3	-2,8	-2,5
Cldn4	-1,1	-2,7	-1,6
Cldn7	-0,3	-2,5	-2,2
Cx3cl1	-1,2	-2,7	-1,5
Cxcl1	-1,2	4,8	6,0
Cxcl14	-1,5	-2,8	-1,3
Cxcl2	0,0	7,3	7,3
Cxcl3	-0,4	4,4	4,7
Cxcl6	-1,3	4,7	6,0
Cxcr2	0,6	4,6	3,9
Cxcr4	1,8	3,9	2,1
Ezr	-0,6	-1,4	-0,8
Fn1	0,5	3,0	2,6
Hrh1	0,0	-1,5	-1,5
Icam1	0,4	1,8	1,4
Icam2	0,3	1,6	1,3
Il18	-0,8	-1,7	-0,9
Il1a	-0,4	3,5	3,9
Il1b	-0,1	4,8	4,9
Il1r1	0,8	3,2	2,4
Il1rn	0,5	5,0	4,5
Il36b	0,9	2,2	1,4
Itga3	-0,5	-1,3	-0,8
Itga4	0,9	1,9	1,0
Itga5	0,5	1,6	1,1
Itga6	-0,6	-1,1	-0,6
Itgb2	1,2	3,4	2,2
Itgb7	0,7	2,5	1,8
Mmp12	0,6	2,7	2,1
Mmp13	-0,8	3,4	4,2
Mmp14	1,2	2,2	1,0
Mmp15	0,3	-1,0	-1,3
Mmp19	0,8	2,0	1,2
Mmp2	0,9	1,5	0,6
Mmp8	-0,3	4,6	4,9
Mmp9	0,1	4,3	4,2
Msn	0,8	2,5	1,7
Myh14	-0,6	-3,0	-2,4
Myl9	-0,6	-2,1	-1,5
Pf4	-0,4	1,7	2,1
Podxl	0,2	1,3	1,1
Sele	0,7	1,8	1,1
Sell	1,4	5,0	3,7
Selp	2,2	3,3	1,1
Selplg	1,7	3,5	1,8
Tnf	0,5	2,5	2,0
Vcam1	0,5	1,6	1,1

B.

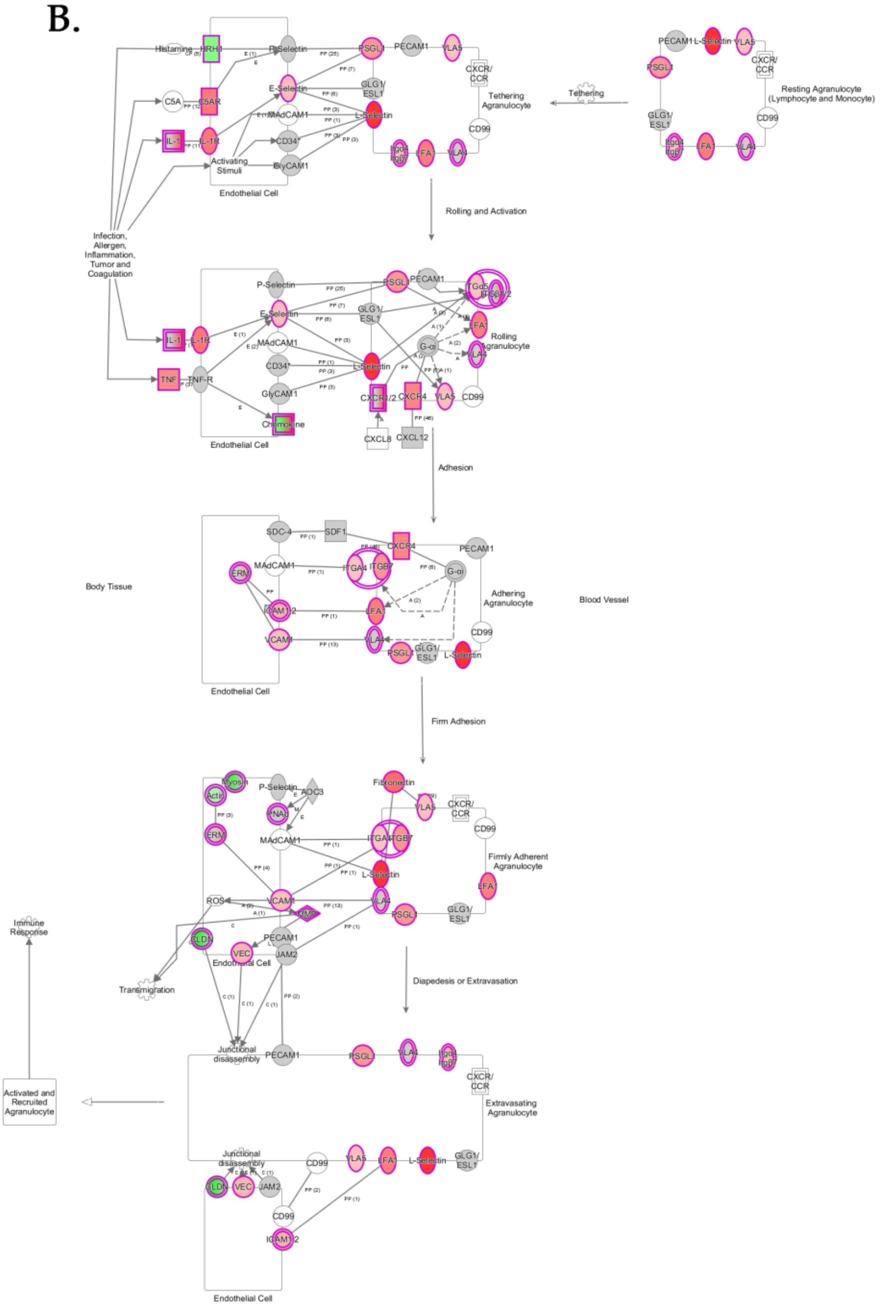

Supplementary Figure 5. Canonical pathway of Agranulocyte adhesion and diapedesis in the TNBS induced rat model of IBD. **A.** Heat map representing expression changes of genes belonging to the "Agranulocyte adhesion and diapedesis" pathway between control, TNBS treated uninflamed and TNBS treated inflamed rat colon samples, where red marks elevation, while blue marks decrease by at least a two-fold in expression level; FDR $p <$ 0.05. Grey background refers no significant alterations. **B.** Visualizing "Agranulocyte adhesion and diapedesis" pathway in the comparison of TNBS treated inflamed to control samples. Red signs show upregulated genes while green signs represent downregulated genes. Genes with no significant changes labelled with grey background, while white signs missing genes from the RNA-Seq analysis. Abbreviations as "UI—C," "I—C" and "I—UI"

represents the TNBS treated—uninflamed vs. control, TNBS-treated—inflamed vs. control and TNBS treated—inflamed vs. TNBS treated—uninflamed comparisons.

Supplementary Table 3 Summary of genes used for the determination of immune and pathway signatures of in vivo model of IBD by ImSig.

	B cells	Interferon	Macrophages	Monocytes	Neutrophils	NK cells	Plasma cells	Proliferation	T cells	Translation
**Total genes in ImSig**	37	66	78	37	47	20	14	99	85	86
**Total genes in ImSig (with Rat homologs)**	**33**	**59**	**73**	**35**	**43**	**10**	**4**	**98**	**78**	**84**
**No. of ImSig genes in user dataset**	31	54	70	30	38	9	4	90	70	67
**No. of ImSig genes after feature selection**	26	48	65	26	36	0	2	84	67	61
**Median correlation of ImSig genes**	0,93	0,34	0,76	0,87	0,78	0,13	0,56	0,75	0,76	0,51
**Median correlation of feature selected ImSig genes**	0,94	0,4	0,8	0,89	0,79	NA	0,86	0,77	0,79	0,56

feature selection at ***r = 0.7***

Supplementary Table 4. List of human and rat gene IDs used for gene signature analysis by ImSig (Part 1).

B cells	
**human ID**	**rat ID**
BANK1	Bank1
HLA-DOB	RT1-DOb
CD72	Cd72
TLR10	Tlr10
CD19	Cd19
TCL1A	Tcl1a
MS4A1	Ms4a1
STAP1	Stap1
BTLA	Btla
CR2	Cr2
FCRL2	Fcrl2
CD180	Cd180
P2RX5	P2rx5
VPREB3	Vpreb3
FCRL1	Fcrl1
FCRL3	Fcrl5
FCRLA	Fcrla
CD79B	Cd79b
SNX22	Snx22
IGHV5-78	
FAM129C	Niban3
FCRL5	Fcrl5
LOC100507616	
CD79A	Cd79a
CCR6	Ccr6
LY9	Ly9
LINC00926	
CD37	Cd37
KIAA0125	
PNOC	Pnoc
CD22	Cd22
PAX5	Pax5
AFF3	Aff3
POU2F2	Pou2f2
S1PR4	S1pr4
BLK	Blk
EBF1	Ebf1

Monocytes	
**human ID**	**rat ID**
PILRA	LOC100910669
PSAP	Psap
LILRB2	
HCK	Hck
THEMIS2	Themis2
EMILIN2	Emilin2
LILRB3	Lilrb3b
FCN1	Fcnb
KIAA0930	RGD1304694
TPP1	Tpp1
LST1	Lst1
AIF1	Aif1
LILRA6	LOC690948
CD93	Cd93
RHOG	Rhog
CD300LF	Cd300lf
FGR	Fgr
CD14	Cd14
TIMP2	Timp2
PLXDC2	Plxdc2
CTSD	Ctsd
HMOX1	Hmox1
C10orf54	Vsir
SLC7A7	Slc7a7
FES	Fes
GNS	LOC100909505
VCAN	Vcan
TGFBI	Tgfbi
NFAM1	Nfam1
PRAM1	Pram1
LRRC25	Lrrc25
GRN	Grn
SERPINA1	Serpina1
PYCARD	Pycard
CD33	
AGTRAP	Agtrap
NOTCH2	Notch2

Neutrophils	
**human ID**	**rat ID**
THBD	Thbd
FPR1	Fpr1
BCL6	Bcl6
TLR2	Tlr2
TNFRSF1A	Tnfrsf1a
RNF149	Rnf149
PLXNC1	Plxnc1
CFLAR	Cflar
PREX1	Prex1
DYSF	Dysf
S100A9	S100a9
S100A8	S100a8
CSF3R	Csf3r
RALB	Ralb
KIAA0247	Susd6
STAT3	Stat3
PHC2	Phc2
IL17RA	Il17ra
GPR97	Adgrg3
MOB3A	Mob3a
CEP19	Cep19
SLC25A37	Slc25a37
LILRA2	
PHF21A	Phf21a
NAMPT	Nampt
SNORD89	
NCF4	Ncf4
TLR4	Tlr4
GLT1D1	Glt1d1
DENND5A	Dennd5a
ACSL1	Acsl1
BASP1	LOC100910172
PADI2	Padi2
LIMK2	Limk2
KCNJ2	Kcnj2
ALPK1	Alpk1
CD97	Adgre5
SSH2	Ssh2
MGAM	LOC679818
STAT5B	Stat5b
IFITM2	Ifitm2
CXCR2	Cxcr2
FAM65B	Ripor2
LINC01002	
AQP9	Aqp9
FCGR2C	
TMEM154	Tmem154

NK cells	
**human ID**	**rat ID**
KIR3DL2	Zbtb21
KIR3DL1	Mroh1
KIR2DS2	
KIR2DS1	
KIR2DS3	
KIR2DL2	
KIR2DL5A	
KIR2DS5	
TBX21	Tbx21
KIR2DL1	
KIR2DL4	
KIR3DL3	Kir3dl1
KIR2DL3	
PRF1	Prf1
KLRC3	Vom1r81
SH2D1B	Sh2d1b
KLRC2	Klrc2
SAMD3	Samd3
KLRC4	
KLRD1	Klrd1

Plasma cells	
**human ID**	**rat ID**
TXNDC5	Txndc5
TNFRSF17	Tnfrsf17
MZB1	Mzb1
IGLV1-44	
IGH	
IGJ	Jchain
IGKC	
IGHG3	
GUSBP11	
IGKV1D-13	
IGLV@	
IGLJ3	
IGLC1	
IGLL3P	

Supplementary Table 5. List of human and rat gene IDs used for gene signature analysis by ImSig (Part 2).

Macrophages	
human ID	rat ID
CECR1	
CTSB	Ctsb
HLA-DRB6	
FCGR2A	LOC498276
TNFSF13B	Tnfsf13b
SLAMF8	Slamf8
IFI30	Ifi30
CCR1	Ccr1
CD163	Cd163
ITGB2	Itgb2
C1QB	C1qb
C3AR1	C3ar1
FCER1G	Fcer1g
TYROBP	Tyrobp
TNFAIP2	Tnfaip2
SLC15A3	Slc15a3
CD74	Cd74
FCGR3B	Fcgr3a
CLEC7A	Clec7a
TRPV2	Trpv2
NCKAP1L	Nckap1l
SPI1	Spi1
CYBB	Cybb
TYMP	Tymp
SNX10	Snx10
VSIG4	Vsig4
HK3	Hk3
IGSF6	Igsf6
MSR1	Msr1
LILRB4	Lilrb4
TBXAS1	Tbxas1
CD300A	Cd300a
TLR8	Tlr8
MNDA	Mnda
FCGR1B	
FPR3	Fpr3
FCGR1A	Fcgr1a
NPL	Npl
CD4	Cd4
LY96	Ly96
MYO1F	Myo1f
CYTH4	Cyth4
CD86	Cd86
LAIR1	Lair1
LAPTM5	Laptm5
PLA2G7	Pla2g7
BCL2A1	Bcl2a1
C2	C2
ADORA3	Adora3
MAN2B1	Man2b1
ARRB2	Arrb2
EMR2	
DPYD	Dpyd
ADAMDEC1	Adamdec1
GPNMB	Gpnmb
MFSD1	Mfsd1
ITGAX	Itgax
CMKLR1	Cmklr1
MS4A7	Ms4a7
TNFRSF1B	Tnfrsf1b
PLEKHO2	Plekho2
MS4A4A	Larp1b
CTSS	Ctss
AOAH	Aoah
ITGAM	Itgam
CSF1R	Csf1r
SLC31A2	Slc31a2
C1QA	C1qa
SCPEP1	Scpep1
C1orf54	
TMEM140	Tmem140
C5AR1	C5ar1
NR1H3	Nr1h3
ATP8B4	Atp8b4
LIPA	Lipa
CD68	Cd68
CCRL2	Ccrl2
SLCO2B1	Slco2b1

T cells	
human ID	rat ID
GIMAP4	Gimap4
CD2	Cd2
ARHGAP9	Arhgap9
IL23A	Il23a
CD48	Cd48
RASSF5	Rassf5
CD52	Cd52
ARHGAP25	Arhgap25
TBC1D10C	Tbc1d10c
NLRC3	Nlrc3
C1orf162	LOC100911379
SP140	Sp140
GPR18	Gpr18
HCST	Hcst
RHOH	Rhoh
GZMK	Gzmk
CORO1A	Coro1a
ITGAL	Itgal
GIMAP7	Gimap7
TRAC	
IL16	Il16
TRAF3IP3	Traf3ip3
EVI2B	Evi2b
DOCK2	Dock2
IL10RA	Il10ra
ARHGAP15	Arhgap15
PRKCH	Prkch
LCP1	Lcp1
CD27	Cd27
FAM26F	Calhm6
DOCK8	Dock8
CD3G	Cd3g
GIMAP2	
NCF1B	
FLI1	Fli1
CXCR6	Cxcr6
SH2D1A	Sh2d1a
PVRIG	Pvrig
CYTIP	Cytip
TRAT1	Trat1
CD3E	Cd3e
GIMAP6	Gimap6
CD96	Cd96
CD3D	Cd3d
FYB	Fyb1
CRTAM	Crtam
CCL19	Ccl19
BIN2	Bin2
PARVG	Parvg
DOCK10	Dock10
TARP	Tarp
KLRB1	Klrb1b
KLHL6	Klhl6
CCR7	Ccr7
CD6	Cd6
UBASH3A	Ubash3a
TRGV9	
PSTPIP1	Pstpip1
IL7R	Il7r
GPR171	Gpr171
EVI2A	Evi2a
APBB1IP	Apbb1ip
AMICA1	Jaml
BTK	Btk
HMHA1	Arhgap45
PTPRCAP	Ptprcap
ITK	Itk
SLA	Sla
GIMAP5	Gimap5
RCSD1	Rcsd1
SASH3	Sash3
FYN	Fyn
TNFRSF9	Tnfrsf9
CD28	Cd28
HVCN1	Hvcn1
CXCL9	Cxcl9
LY86	Ly86
RGS18	Rgs18
TRGC2	
DPEP2	Dpep2
SIRPG	
CD8A	Cd8a
ICOS	Icos
GAB3	
GMFG	Gmfg

Proliferation	
human ID	rat ID
GINS2	Gins2
UHRF1	Uhrf1
ZWILCH	Zwilch
FANCI	Fanci
RAD51AP1	Rad51ap1
SMC2	Smc2
GINS1	Gins1
NCAPG2	Ncapg2
RAD51	Rad51
NDC80	Ndc80
MCM6	Mcm6
DTL	Dtl
HMMR	Hmmr
EZH2	Ezh2
KIAA0101	Pclaf
MAD2L1	Mad2l1
PBK	Pbk
CDK1	Cdk1
TOP2A	Top2a
CCNB1	Ccnb1
ZWINT	Zwint
CENPE	Cenpe
ANLN	Anln
CENPF	Cenpf
CCNA2	Ccna2
KIF20A	Kif20a
BUB1B	Bub1b
TTK	Ttk
PTTG1	Pttg1
NUSAP1	Nusap1
NUF2	Nuf2
RACGAP1	Racgap1
MELK	Melk
RRM1	Rrm1
TCF19	Tcf19
CDT1	Cdt1
POLE2	Pole2
TK1	Tk1
MCM4	Mcm4
GMNN	Gmnn
MCM2	Mcm2
MND1	Mnd1
CDC20	Cdc20
DEPDC1B	Depdc1b
PCNA	Pcna
CDC6	Cdc6
FOXM1	Foxm1
RRM2	Rrm2
TYMS	Tyms
TRIP13	Trip13
MTFR2	Mtfr2
CENPL	Cenpl
BIRC5	Birc5
CKS1B	Cks1b
MCM10	Mcm10
SPC25	Spc25
E2F8	E2f8
CDCA2	Cdca2
CDKN3	Cdkn3
CDCA3	Cdca3
CENPA	Cenpa
UBE2C	Ube2c
OIP5	Oip5
KIF11	Kif11
CCNB2	Ccnb2
NEK2	Nek2
STIL	Stil
ECT2	Ect2
AURKA	Aurka
KIF15	Kif15
KIF2C	Kif2c
KIF18B	Kif18b
KIF14	Kif14
TPX2	Tpx2
FAM72C	
DLGAP5	Dlgap5
ASPM	Aspm
DEPDC1	Depdc1
KIF4A	Kif4a
HMGB3	LOC688583
CDCA8	Cdca8
PLK4	Plk4
CCNE2	Ccne2
CDCA7	Cdca7
SHCBP1	Shcbp1
STMN1	Stmn1
DONSON	Donson
PARPBP	Parpbp
SKA1	Ska1
FBXO5	Fbxo5
MKI67	Mki67
CEP55	Cep55
NCAPG	Ncapg
POLQ	Polq
AURKB	Aurkb
CASC5	Knl1
HJURP	Hjurp
CDCA5	LOC686151
BUB1	Bub1

Interferon	
human ID	rat ID
STAT2	Stat2
GBP4	Gbp4
IRF7	Irf7
IFI44L	Ifi44l
ISG15	Isg15
SP110	Sp110
IFI44	Ifi44
XAF1	Xaf1
STAT1	Stat1
DTX3L	Dtx3l
OAS3	Oas3
MX1	Mx2
SAMD9L	Samd9l
IFIH1	Ifih1
PARP9	Parp9
IFIT3	Ifit3
TRAFD1	Trafd1
C5orf56	
BST2	Bst2
IFITM1	Ifitm1
C19orf66	Shfl
RNF213	Rnf213
IFI16	Mnda
IRF9	Irf9
TRIM22	
RTP4	Rtp4
BATF2	Batf2
TAP1	Tap1
GBP1	Gbp1
PSMB9	Psmb9
MX2	RGD1308751
ZNFX1	Znfx1
LAMP3	Lamp3
IFIT5	
SAMD9	Samd9
IFI35	Ifi35
EPSTI1	Epsti1
PARP12	Parp12
IFIT2	Ifit2
CMPK2	Cmpk2
UBE2L6	Ube2l6
OAS2	Oas2
PARP14	Parp14
PHF11	Phf11
SHISA5	Shisa5
FBXO6	Fbxo6
PARP10	Parp10
IFI6	
APOL6	
USP18	Usp18
DDX60	Ddx60
LAP3	Lap3
HERC6	Herc6
RSAD2	Rsad2
IFIT1	Ifit1bl
PML	Pml
TRIM5	Trim5
APOL1	
DHX58	Dhx58
HSH2D	Hsh2d
SIGLEC1	Siglec1
DDX58	Ddx58
HELZ2	Helz2
HERC5	
OASL	Oasl
TRIM21	Trim21

Translation	
human ID	rat ID
RPS3A	LOC100365839
FAU	LOC687780
RPS23	Rps23
RPL18A	Rpl18a
EIF3F	Eif3f
RPL24	Rpl24
RPS15A	Rps15a
SNHG8	
RPL11	Rpl11
RPL15	Rpl15
PFDN5	Pfdn5
EIF3E	Eif3el1
RPS25	Rps25
RPL31	Rpl31
RPL18	Rpl18
EEF1B2	Eef1b2
RPL5	Rpl5
RPS9	Rps9
RPL10	Rpl10
RPS27A	Rps27a-ps1
RPL7A	Rpl7a
RPSA	Rpsa
RPS19	Rps19
RPS6	Rps6
RPLP2	Rplp2
RPL13A	Rpl13a
RPS13	Rps13
RPL6	Rpl6
RPS18	Rps18l1
RPS15	Rps15
RPS28	Rps28
RPL9	Rpl9
RPL32	LOC680959
RPL23	Rpl23
RPS3	Rps3
RPS5	Rps5
RPL17	Rpl17
RPS14	Rps14
RPL12	Rpl12
RPL3	Rpl3
RPL21	Rpl21
NACA	Naca
RPL13	Rpl13
RPL34	Rpl34l1
RPL14	Rpl14
RPL29	Rpl29
RPS8	Rps8
RPS16	Rps16
EIF3H	LOC100911110
RPL35A	Rpl35al1
RPS10	Rps10l1
RPL19	Rpl19
EIF3D	Eif3d
RPL38	Pramef27
RPS7	Rps7
RPS17	Rsl1
RPL27	Rpl27
RPL37	Rpl37
RPL35	Rpl35
RPS2	Rps2
RPL36A	LOC100912182
EEF1G	Eef1g
GNB2L1	Rack1
EEF1D	Eef1d
RPL7	Rpl7
SNHG6	
RPS20	Rps20
RPL30	Rpl30
RPL28	Rpl28
RPS29	Rps29
RPL8	Rpl8
RPS21	Rps21
RPL23A	RGD1564606
RPL22	Rpl22
RPL27A	Rpl27a
RPS11	Rps11
RPL37A	Rpl37a
RPL10L	Rpl10l
RPLP0	Rplp0
RPL4	Rpl4
EIF3G	Eif3g
SNRPD2	Snrpd2
RPL39	Rpl39
EEF1A1	Eef1a1
EIF3K	Eif3k
UXT	Uxt
